# Acute thalamic connectivity precedes chronic post-concussive symptoms in mild traumatic brain injury

**DOI:** 10.1093/brain/awad056

**Published:** 2023-02-22

**Authors:** Rebecca E Woodrow, Stefan Winzeck, Andrea I Luppi, Isaac R Kelleher-Unger, Lennart R B Spindler, J T Lindsay Wilson, Virginia F J Newcombe, Jonathan P Coles, CENTER-TBI MRI Substudy Participants and Investigators, Krisztina Amrein, Nada Andelic, Lasse Andreassen, Audny Anke, Philippe Azouvi, Bo-Michael Bellander, Habib Benali, Andras Buki, Alessio Caccioppola, Emiliana Calappi, Marco Carbonara, Giuseppe Citerio, Hans Clusmann, Mark Coburn, Jonathan Coles, Marta Correia, Endre Czeiter, Véronique De Keyser, Vincent Degos, Bart Depreitere, Live Eikenes, Erzsébet Ezer, Kelly Foks, Shirin Frisvold, Alexandre Ghuysen, Damien Galanaud, Ben Glocker, Asta Haberg, Iain Haitsma, Eirik Helseth, Peter J Hutchinson, Evgenios Kornaropoulos, Noémi Kovács, Ana Kowark, Steven Laureys, Didier Ledoux, Hester Lingsma, Andrew I R Maas, Geoffrey Manley, David K Menon, Tomas Menovsky, Benoit Misset, Visakh Muraleedharan, Ingeborg Nakken, Virginia Newcombe, Wibeke Nordhøy, József Nyirádi, Fabrizio Ortolano, Paul M Parizel, Vincent Perlbarg, Paolo Persona, Wilco Peul, Jussi P Posti, Louis Puybasset, Sophie Richter, Cecilie Roe, Olav Roise, Rolf Rossaint, Sandra Rossi, Daniel Rueckert, Toril Skandsen, Abayomi Sorinola, Emmanuel Stamatakis, Ewout W Steyerberg, Nino Stocchetti, Riikka Takala, Viktória Tamás, Olli Tenovuo, Zoltán Vámos, Gregory Van der Steen, Wim Van Hecke, Thijs Vande Vyvere, Jan Verheyden, Anne Vik, Victor Volovici, Lars T Westlye, Guy Williams, Stefan Winzeck, Peter Ylén, Tommaso Zoerle, David K Menon, Emmanuel A Stamatakis

**Affiliations:** University Division of Anaesthesia, University of Cambridge, Addenbrooke’s Hospital, Cambridge CB2 0SP, UK; Department of Clinical Neurosciences, University of Cambridge, Addenbrooke’s Hospital, Cambridge CB2 0SP, UK; University Division of Anaesthesia, University of Cambridge, Addenbrooke’s Hospital, Cambridge CB2 0SP, UK; BioMedIA Group, Department of Computing, Imperial College, London SW7 2RH, UK; University Division of Anaesthesia, University of Cambridge, Addenbrooke’s Hospital, Cambridge CB2 0SP, UK; Department of Clinical Neurosciences, University of Cambridge, Addenbrooke’s Hospital, Cambridge CB2 0SP, UK; The Alan Turing Institute, London NW1 2DB, UK; University Division of Anaesthesia, University of Cambridge, Addenbrooke’s Hospital, Cambridge CB2 0SP, UK; Department of Clinical Neurosciences, University of Cambridge, Addenbrooke’s Hospital, Cambridge CB2 0SP, UK; University Division of Anaesthesia, University of Cambridge, Addenbrooke’s Hospital, Cambridge CB2 0SP, UK; Department of Clinical Neurosciences, University of Cambridge, Addenbrooke’s Hospital, Cambridge CB2 0SP, UK; Division of Psychology, University of Stirling, Stirling FK9 4LA, UK; University Division of Anaesthesia, University of Cambridge, Addenbrooke’s Hospital, Cambridge CB2 0SP, UK; University Division of Anaesthesia, University of Cambridge, Addenbrooke’s Hospital, Cambridge CB2 0SP, UK; University Division of Anaesthesia, University of Cambridge, Addenbrooke’s Hospital, Cambridge CB2 0SP, UK; Wolfson Brain Imaging Centre, University of Cambridge, Cambridge Biomedical Campus, Cambridge CB2 0QQ, UK; University Division of Anaesthesia, University of Cambridge, Addenbrooke’s Hospital, Cambridge CB2 0SP, UK

**Keywords:** thalamus, mild traumatic brain injury, functional connectivity, postconcussive symptoms, resting-state fMRI

## Abstract

Chronic post-concussive symptoms are common after mild traumatic brain injury (mTBI) and are difficult to predict or treat. Thalamic functional integrity is particularly vulnerable in mTBI and may be related to long-term outcomes but requires further investigation.

We compared structural MRI and resting state functional MRI in 108 patients with a Glasgow Coma Scale (GCS) of 13–15 and normal CT, and 76 controls. We examined whether acute changes in thalamic functional connectivity were early markers for persistent symptoms and explored neurochemical associations of our findings using PET data.

Of the mTBI cohort, 47% showed incomplete recovery 6 months post-injury. Despite the absence of structural changes, we found acute thalamic hyperconnectivity in mTBI, with specific vulnerabilities of individual thalamic nuclei. Acute fMRI markers differentiated those with chronic post-concussive symptoms, with time- and outcome-dependent relationships in a sub-cohort followed longitudinally. Moreover, emotional and cognitive symptoms were associated with changes in thalamic functional connectivity to known serotonergic and noradrenergic targets, respectively.

Our findings suggest that chronic symptoms can have a basis in early thalamic pathophysiology. This may aid identification of patients at risk of chronic post-concussive symptoms following mTBI, provide a basis for development of new therapies and facilitate precision medicine application of these therapies.

## Teaser

Woodrow *et al*. show that acute thalamic connectivity changes precede long-term post-concussive symptoms, even in the mildest traumatic brain injury. The changes are related to specific symptoms and key neurotransmitter systems, providing prognostic and therapeutic opportunities.

## Introduction

Despite its label, mild traumatic brain injury (mTBI) is commonly associated with persistent symptoms and incomplete recovery. The symptoms include depression, cognitive impairment, headaches and fatigue, whereby over half of mTBI patients report three or more symptoms at 6 months post-injury.^1^ Despite this, clinical care and outcome prognostication in mTBI are poor. A recent study of over 200 mTBI cases showed that, whilst clinicians predicted 90% would fully recover by 6 months, only 50% achieved full functional and symptomatic recovery.^2^ Thus, many mTBI patients may not be adequately assessed and cared for post-injury, particularly beyond the acute phase of their illness. Moreover, current predictive models of functional outcome in mild TBI perform poorly,^3^ and we have few effective and systematic therapies, or indeed, therapy targets, for treatment of these patients.

This combination of over-optimistic and imprecise prognoses, therapeutic paucity and frequent unfavourable outcome provide an unsatisfactory context for care of patients with mTBI. This is a particularly pertinent issue, given the rising number of mTBI cases, which represent ∼90% of all TBI, affecting ageing populations who experience falls, rising numbers of road traffic collisions in low- and middle-income countries and widespread concerns regarding long-term effects of sports-related concussion.^4^ One way to better understand disease pathophysiology is to investigate brain–behaviour relationships, which can lead to improved prognostication algorithms, diagnostic criteria and more informed treatment strategies.

The thalamus is a particularly under-investigated brain region in mTBI. It holds a pivotal role in information transfer between motor, sensory and associative cortical regions and in coordinating complex cognition across the cortex and its networks.^5^ It is particularly susceptible to acute injury-induced damage^6,7^ and shows evidence for secondary damage due to delayed transneuronal degeneration.^8^ Even in the absence of overt structural damage following experimental diffuse TBI, the thalamus shows persistent and unresolved histopathological evidence of neuronal injury.^9^

Thalamic dysfunction has long been implicated in common post-concussive symptoms such as headache,^10^ sleep disturbances,^11^ fatigue^12^ and cognition.^13^ Furthermore, morphological thalamic damage in severe TBI is predictive of long-term functional and neuropsychological outcomes^14^ and chronic fatigue.^15^ In mTBI cohorts, the thalamus shows prolonged volumetric loss associated with greater symptom reporting^16^ and lower cerebral blood flow in the chronic phase associated with poorer cognitive performance.^13^ Thus, the thalamus could be an important region of interest in pathogenesis and prognosis following mTBI.^17^

However, signs of structural damage on CT or structural MRI are uncommon in the acute phase mTBI populations.^18^ While late structural MRI does demonstrate changes in the post-acute phases,^15,16,19^ imaging at late time-points has no prognostic application and may represent the consequences of pathophysiology rather than mapping injury processes. Functional imaging provides a more sensitive means of investigating earlier thalamic damage and its relationship to outcome, where resting state functional MRI (rs-fMRI) allows exploration of wider thalamic function and symptoms than specific cognitive domains in task-based fMRI.

Indeed, a few studies have previously investigated thalamic functional connectivity (temporal correspondence of two time-courses of activity) after mTBI in the acute/subacute phase and suggest injury-induced thalamic hyperconnectivity. This increased connectivity is widespread and found sub-acutely in anterior prefrontal cortex and supramarginal gyrus^20^ and acutely in posterior cingulate, dorsal anterior cingulate cortex, bilateral medial temporal regions, the default mode network and primary sensory regions.^21^ Further evidence suggests such widespread changes may be due to a breakdown in thalamocortical communication, evidenced by subacute reductions in thalamic topographical efficiency.^22^ Other small studies have correlated thalamic functional change with symptomatology in mTBI. Increased spread and asymmetry of thalamic resting-state networks have been linked to increased concurrent subacute depression, post-concussive symptoms and impaired cognitive performance,^23^ while increasing functional connectivity of the thalamus and dorsal attention network over 6 weeks–4 months correlated with decreases in self-reported pain and post-concussive symptoms.^24^ These reports support a relationship between widespread thalamic hyperconnectivity and post-concussive symptoms, potentially driven by selective thalamic vulnerability.

Many previous studies, however, included patients with pre-existing risk factors (such as pre-injury psychiatric disease) for post-TBI symptoms, and it remains impossible to disentangle the neuroimaging consequences of these factors from those due to TBI. Furthermore, none of these studies relate imaging-derived measures associated with symptoms to their neurochemical basis or potential therapeutic targets. This is an unmet need, as current treatments for post-concussive symptoms lack both evidence-based support and a clear biological framework.^25^ Current literature lacks studies with sufficient sample sizes and longitudinal follow-up and has not yet investigated the role of biologically-relevant subdivisions of the thalamus or the neurochemical associations of such connectivity changes. Individual nuclei have different biological properties, primary functions and cortical connectivity. They may therefore have differential prognostic specificity and therapeutic relevance.

This manuscript reports on thalamic changes after mTBI, with the hypothesis that nuclei-specific functional hyperconnectivity is present acutely after injury with relevance for persistent symptoms. Additionally, specific symptoms may show distinguishing profiles of acute connectivity change, with the exploratory hypothesis that potential underlying neurochemical relationships may guide future therapeutic opportunities. These novel methodological approaches, in combination with granular data from a state-of-the-art clinical study (CENTER-TBI -Collaborative European NeuroTrauma Effectiveness Research in TBI^26^), presents the neuroanatomical basis, neurochemical associations, prognostic implications and therapeutic opportunities of understanding abnormalities in thalamic functional connectivity following mTBI.

## Materials and methods

### Study design

Data for the analyses in this manuscript were obtained from subjects recruited to the MRI sub-study of CENTER-TBI between December 2014 and December 2017 (https://clinicaltrials.gov/ct2/show/NCT02210221), version CENTER CORE 3.0. Ethical approval was obtained in accordance with relevant laws and regulations for each recruiting site, and informed consent was given by each participant either directly or by a legal representative/next of kin. Further details of sites and ethical approvals can be found at https://www.center-tbi.eu/project/ethical-approval.

Inclusion criteria were that subjects were aged 18–70 years with no medical history of previous concussion or neuropsychiatric disease. We included 108 patients who additionally sustained a mTBI [Glasgow Coma Scale (GCS) 13–15], required a head CT according to local criteria on initial presentation, showed no CT abnormalities and had both T_1_-weighted MRI and rsfMRI in the acute phase post-injury. Matched healthy controls (*n* = 76) were recruited from the same centres as patients and contemporaneously imaged on the same MRI systems. Additionally, a serial imaging cohort of *n* = 31 patients had structural and functional imaging at 6 and 12 months post injury and thus were followed longitudinally. These are summarized in a consort diagram (Supplementary Fig. 1). Demographic information for all mTBI patients according to the above criteria, regardless of whether acute imaging was collected, is shown in Supplementary Table 1 to demonstrate similarity of our cohort to the wider mTBI population.

### Outcome groups

Six-month outcomes assessed functional and symptomatic recovery using the Glasgow Outcome Scale–Extended^27^ (GOSE) and Rivermead Postconcussion Symptom Questionnaire^28^ (RPQ). The GOSE rates patient function into eight categories from death (1) to upper-good recovery (8), whereas the RPQ is a self-report measure of experienced severity of 16 most-commonly cited post-concussion symptoms compared with pre-injury levels, on a five-point scale from 0 indicating ‘not experienced’ to 4 as ‘a severe problem’. These measures were binarized to ‘complete’ (GOSE-8) versus ‘incomplete’ (GOSE ≤7) recovery and post-concussive symptom-(PCS) positive or negative. PCS groups were defined in accordance with the International Classification of Diseases 10th Revision, whereby a rating of 2 (a mild problem) or above is a ‘reported’ symptom and subjects reported at least three of the specified symptoms. Post-concussive symptoms were further explored using the three-factor structure of the RPQ, encompassing cognitive, emotional and somatic domains.^29^ Groups were defined based on those who presented (≥1) or did not present (<1) that factor by taking a mean of the relevant RPQ items. These arguably lenient groupings were used due to the mild nature of the cohort to ensure any presenting symptoms would be captured, and sample sizes were suitable for group comparisons.

### Imaging acquisition and preprocessing

Acquisition protocols for CENTER-TBI are described in the central CENTER-TBI resources at https://www.center-tbi.eu/project/mri-study-protocols. Importantly, structural (T_1_-weighted) and functional (rsfMRI) imaging were performed during the acute phase after injury. Preprocessing of T_1_-weighted and rsfMRI data was largely performed using fmriprep^30^ (v1.5.4), which combines best-judged aspects of different software for standardized and freely accessible preprocessing. This is further described in the Supplementary material in their automated boilerplate. Functional data were then spatially smoothed (6 mm gaussian kernel) and denoised via signal regression of fmriprep-derived parameters (white matter, CSF, rigid-body head motion 6 degrees of freedom, temporal high pass filter) using Python package Nilearn.^31^ Any volumes identified as motion outliers by fmriprep were removed from the data (denoising quality control can be found in the Supplementary material), but no individual subjects were removed as all exceeded 4 min of data acquisition after scrubbing. Finally, some participants’ functional data had a higher number of volumes than the group mode (*n* = 164); therefore, any volumes over this were removed from the end of acquisition for group-level analysis.

### Thalamus subdivisions

The left and right thalamus and seven thalamic nuclei per hemisphere (*n* = 16 regions of interest, ROIs) were investigated using the probabilistic atlas defined by Najdenovska and colleagues.^32^ This was obtained from a large and healthy sample, has proven a successful substitution for individually-segmented thalamic nuclei,^32^ and more than seven subdivisions seemed unfeasible, given the spatial resolution of fMRI data to give sufficient specificity in clinical populations. Atlas standard space was transformed into preprocessed standard space using affine transformation with 12 degrees of freedom, a 180 degree search angle and trilinear interpolation and applied to thalamic maximum probability masks. These were re-binarized at values >0.5 to avoid transformation-induced overlaps, which could impact results. Nuclei are shown in Supplementary Fig. 7.

### Thalamic volume

For volume extraction, each raw T_1_-weighted scan was first corrected for scanner bias field inhomogeneities^33^ and spatially normalized to the MNI ICBM152 T_1_-weighted template corresponding to thalamic atlas space^34^ via affine and non-linear registration.^35^ To estimate spatial normalization quality, the zero-normalized cross correlation (ZNCC) was computed between aligned T_1_-weighted scans and the T_1_-weighted template image:


(1) $$ZNCC=\frac{1}{N}\sum_{x,y}\frac{\;(x_{i}\;-\;\mu_{x})\;(y_{i}\;-\;\mu_{y})}{\sigma_{x}\sigma_{y}\;}\;$$

with *N* being the number of voxels within the brain mask of image *x* (the projected T_1_-weighted scans) and image *y* (the T_1_-weighted template), and *μ* and *σ* representing the mean and standard deviation respectively. A high ZNCC value corresponds to high similarity between image intensities and indicates a successful spatial alignment between scans.

The inverse of the found transformations were used to project the thalamus atlas with nearest neighbour interpolation from Montreal Neurological Institute (MNI) template space to each subject’s individual T_1_-weighted space. Volumes of thalamus (left and right) and its nuclei were computed by summing the voxels of the back-projected atlas regions and multiplying by the single-voxel volume. Eventually, thalamic volumes were normalized by the total brain volume, estimated via automated brain extraction.^36^

### Thalamic functional connectivity

Three lines of thalamic functional connectivity were investigated. First, average thalamocortical connectivity was investigated using the CONN toolbox v.20.b,^37^ as previous work in mTBI has found widespread functional alterations across the cortex. For each participant, this provided beta maps of ROI-to-voxel connectivity for all (*n* = 16) thalamic ROIs and a mean was calculated within each individual's cortical grey matter mask. Secondly, functional connectivity between thalamic ROIs was calculated by correlating each pair of average time-courses (first five volumes removed) to obtain a correlation coefficient and applying Fisher's *r*-to-*z* transform. Each of *n* = 184 subjects therefore had a 16 × 16 matrix of within-thalamus connectivity values. Finally, local brain-wide functional connectivity changes were assessed using previously-calculated beta maps and studied for voxel-wise connectivity differences between groups using SPM12.^38^

### Association to neurotransmitter systems

Neurotransmitter systems become strongly dysregulated following injury.^39^ Consequently, to better understand potential underpinnings of altered connectivity and characterize possible therapeutic avenues for chronic symptomatology, we explored whether voxel-wise clusters of significant change from group comparisons might be related to densities of specific neurotransmitter receptors and/or transporters.

Receptor densities were estimated using group-averaged PET receptor/transporter maps obtained from healthy volunteers for a total of 18 receptors and transporters across nine neurotransmitter systems, as detailed in recent work by Hansen and colleagues.^40^ These included dopamine (D1, D2, DAT), norepinephrine (NET), serotonin (5-HT1A, 5-HT1B, 5-HT2A, 5-HT4, 5-HT6), acetylcholine (*α*4*β*2, M1, VAChT), glutamate (mGluR5), GABA (GABA-A), histamine (H3), cannabinoid (CB1) and opioid (MOR). Volumetric PET images were registered to the MNI-ICBM 152 nonlinear 2009 (version c, asymmetric) template, averaged across participants within each study and then parcellated, and receptors/transporters with more than one mean image of the same tracer (5-HT1b, D2, VAChT) were combined using a weighted average. See Hansen *et al*.^40^ for detailed information about each map.

Both the PET and statistical maps of seed-to-voxel correlation *t*-scores were then parcellated into discrete cortical regions according to the recent local-global cortical functional atlas of Schaefer^41^ scales 100 and 200, and the multimodal cortical parcellation of Glasser^42^ with 360 cortical regions.

### Blood biomarkers

To assess the clinical value of our imaging-derived thalamic markers of outcome, we compared values of six common blood biomarkers of injury between the outcome groups of GOSE and PCS as defined above. These were, neuron-specific enolase (NSE), S-100 calcium-binding protein B (S100B), glial fibrillary acidic protein (GFAP), Tau, ubiquitin C-terminal hydrolase-L1 (UCH-L1) and neurofilament light chain (NFL). These values were obtained from CENTER-TBI (CORE v3.0) and collected within 30 days post-injury for inclusion.

### Statistical analyses

All statistical analyses were conducted using R (v.4.1.2) at a false discovery rate (FDR)-corrected significance level of *P* ≤ 0.05 unless otherwise stated. Missing demographic data typical of large datasets were handled by multiple imputation using the Multivariate Imputation by Chained Equations algorithm^43^ with *n* = 5 imputations. This modelled missing data using existing age and sex with a logistic regression model for binary data (sex; *n* = 2 controls) applied within groups to avoid potential group effects.

Control and mTBI groups were initially compared for two-tailed differences in age (Fisher's exact) and sex (chi-squared). Tests were chosen to account for the categorical nature of recruitment in CENTER-TBI protocols,^26^ which aim to combat possible differences in admission rates during study recruitment. However, age is hereafter treated as a continuous covariate in all statistical analyses. Outcome groups were also compared for these covariates, and additionally for time since scan of injury (independent samples *t*-test) and baseline GCS (Fisher's exact).

To address issues of multicentre acquisition associated with large datasets,^44^ efforts were made to statistically harmonize each data type across *n* = 14 sites/scanners using NeuroCombat.^45^ This recently validated empirical Bayesian method has successfully been applied in previous diffusion imaging,^45,46^ cortical thickness^47^ and rsfMRI studies,^48–50^ and models an expected biological value such as volume or connectivity as a linear combination of biological variables and site differences, whereby error is modulated by site-specific factors. Importantly, clinical group, age and sex were included in the model as covariates to avoid overcorrection and preserve these important biological trends. Harmonization was applied prior to statistical analysis on each imaging-derived value type individually.

For thalamic volume, average thalamocortical functional conectivity, and within-thalamus functional conectivity, each variable was compared between cohort groups (controls versus patients) using a linear model with type III sum of squares to assess the significance of group membership, while controlling for covariates of sex and age. Thalamic volumes were additionally controlled for spatial normalization quality (ZNCC) within the model. Variables with significant differences were then similarly compared between outcome groups, further accounting for age, sex, time since injury and baseline GCS in the linear model. Additionally, acute blood biomarker values were compared between outcome groups using these same statistical criteria.

Final mTBI versus control comparisons of voxel-wise functional connectivity used SPM12^38^ and ran a one-sample *t*-test (family-wise error *P* = 0.01, implicit mask) to establish the most implicated voxels across participants’ beta-maps. Second-level analysis was constrained by the one-sample results’ mask, and we ran two-sample *t*-tests between control and mTBI groups. These tests were conducted with thresholds set at *P* < 0.001 (uncorrected) at the voxel level and family-wise error-corrected *P* < 0.05 at the cluster level, repeated for all *n* = 16 thalamic ROIs. Results informed further investigation of functional connectivity differences between outcome groups according to the same statistical criteria.

Seed-to-voxel *t*-maps with significant clusters were further parcellated and correlated with *z*-scored PET maps to assess their spatial correspondence. This focused on three nuclei of interest identified previously and with significant clusters of group differences. The statistical significance of correlations was tested against a rigorous null model that considers the spatial dependency of the data by using spatial autocorrelation-preserving permutation tests, termed spin tests.^51,52^ Parcel coordinates were projected onto the spherical surface and then randomly rotated and original parcels were reassigned the value of the closest rotated parcel. This procedure was performed with 10 000 repetitions, thereby obtaining a null distribution with preserved spatial autocorrelation. This spin test embodies the null hypothesis that neurotransmitter density and thalamic seed-based functional connectivity are spatially correlated with each other only because of inherent spatial autocorrelation. Significantly associated PET maps at the mTBI-Control level were taken forward to comparisons between outcome groups and only in those maps where significant voxel-wise differences were found. All *P*-values were corrected for multiple comparisons using FDR-correction within-test and required replication across all three parcellation schemes for additional robustness of our results.

Our final analyses investigated longitudinal changes in the serial imaging cohort. These were compared in demographic characteristics and imaging-derived variables to the non-follow-up cohort as described above to ensure continuity between acute and longitudinal findings. All data were preprocessed and analysed with covariates as before, calculating thalamic volume and average thalamocortical connectivity from each nucleus but without statistical harmonization for site differences due to smaller sample size, reducing its success across the *n* = 4 sites present. These were compared to controls as previously described. Based on the hypothesis that longitudinal connectivity changes may depend on outcome,^53^ significant variables were further compared to PCS status using a two-way mixed-ANOVA, where PCS status was defined based on previously described criteria being met at 6 and/or 12 months (between-subjects), and time of imaging was acute or 12 months post-injury (within-subjects). Significant interaction effects were further explored for effects of PCS group, with a *post hoc* within-subjects linear model with covariates of age, sex, initial GCS and time between acute and 12-month imaging.

### Data availability

Raw CENTER-TBI data can be accessed by application at https://www.center-tbi.eu/data. Derived data and code can be made available upon reasonable request.

## Results

### Demographic, clinical and outcome characteristics

Patient and control groups did not differ in age [*X*^2^(1) = 2.2, *P* = 0.34], sex [*X*^2^(1) = 0.2, *P* = 0.64] or spatial normalization quality of structural imaging [*F*(1,180) = 1.07, *P* = 0.30]. Imaging was performed at a mean of 13.74 (SD 9.86) days post-injury, and 6-month outcomes collected at a mean of 197 (SD 33.0) days post-injury.

Of the patient cohort, 45.2% of participants presented incomplete 6-month recovery (GOSE ≤7), and 31.6% were classified as PCS + . Group membership on outcome categories were not related to age [*X*^2^(1) = 0.6, *P* = 0.75; *X*^2^(1) = 0.2, *P* = 0.90], sex [*X*^2^(1) = 0.1, *P* = 0.76; *X*^2^(1) = 0.001, *P* = 0.97], time since injury for scan [*t*(104) = 1.2, *P* = 0.23; *t*(96) = 0.6, *P* = 0.54] or baseline GCS (Fisher's exact *P* = 0.79; *P* = 0.69) for GOSE or PCS, respectively. The PCS+ group was largely a subset of the incomplete functional recovery group, as *n* = 28/31 PCS+ were also classified as GOSE ≤ 7. Thus, *n* = 51 participants were not functionally and/or symptomatically recovered at 6 months, representing 47.2% of the cohort. Further demographic, injury-specific and outcome information can be seen in Table 1.

**Table 1:** Baseline demographic, injury and outcome measures by clinical group

	Control (*n* = 76) *n* (%)	mTBI (*n* = 108) *n* (%)
**Age**		
18–35	26 (34.2)	29 (26.9)
36–55	36 (47.4)	50 (46.2)
55–70	14 (18.4)	29 (26.9)
**Sex**		
Male	46 (60.5)	69 (63.9)
Female	30 (39.5)	39 (36.1)
**Glasgow Coma Score**		
15	–	88 (81.5)
14	–	19 (17.6)
13	–	1 (0.9)
**Injury Cause**		
Road Traffic Incident	–	51 (47.2)
Incidental Fall	–	38 (35.2)
Other Non-intentional injury	–	7 (6.5)
Violence/Assault	–	7 (6.5)
Act of Mass Violence	–	1 (0.9)
Unknown	–	4 (3.7)
**Strata**		
Emergency Room	–	48 (44.4)
Admission	–	60 (55.6)
**6-Month GOSE**		***n* = 106**
Score (*n*)	–	1 (1); 4 (1); 5 (2); 6 (18); 7 (26); 8 (58)
Complete	–	58 (54.7)
Incomplete	–	48 (45.2)
**6-Month PCS**		***n* = 98**
PCS+	–	31 (31.6)
PCS−	–	67 (68.4)

Most prevalent symptoms reported at greater than pre-injury levels were fatigue (*n* = 34/98), poor concentration (*n* = 25/98) and headaches (*n* = 25/98) (Supplementary Fig. 2). Change in post-concussive symptom severity from baseline to 6-months post-injury are shown in Supplementary Fig. 3. Patients were further split into those with or without cognitive (Cog+ *n* = 38; Cog− *n* = 60), emotional (Emo+ *n* = 38; Emo− *n* = 60) or somatic symptoms (Som+ *n* = 23; Som− *n* = 75). Group membership was not related to age [*Χ*^2^(1) = 0.4, *P* = 0.82; *X*^2^(1) = 0.2, *P* = 0.91; *X*^2^(1) = 1.1, *P* = 0.59], sex [*X*^2^(1) = 0.4, *P* = 0.54; *X*^2^(1) = 2.2, *P* = 0.14; *X*^2^(1) = 1.9, *P* = 0.17], time since injury for scan [*t*(96) = 0.7, *P* = 0.48; *t*(96) = 0.3, *P* = 0.79; *t*(96) = 1.2, *P* = 0.25] or baseline GCS (Fisher's exact *P* = 1; *P* = 1; *P* = 0.81) in cognitive, emotional or somatic groups, respectively. The cognitive and emotional sub-groups showed significant overlap of patient inclusion [*X*^2^(1) = 42, *P* < 0.001; *n* = 30 with both cognitive and emotional symptoms] but are investigated here as separate phenotypes. More detail on these sub-groups is given in Supplementary Fig. 4.

### Functional, but not structural, thalamic changes are seen in the acute phase of mTBI

**Figure 1:**
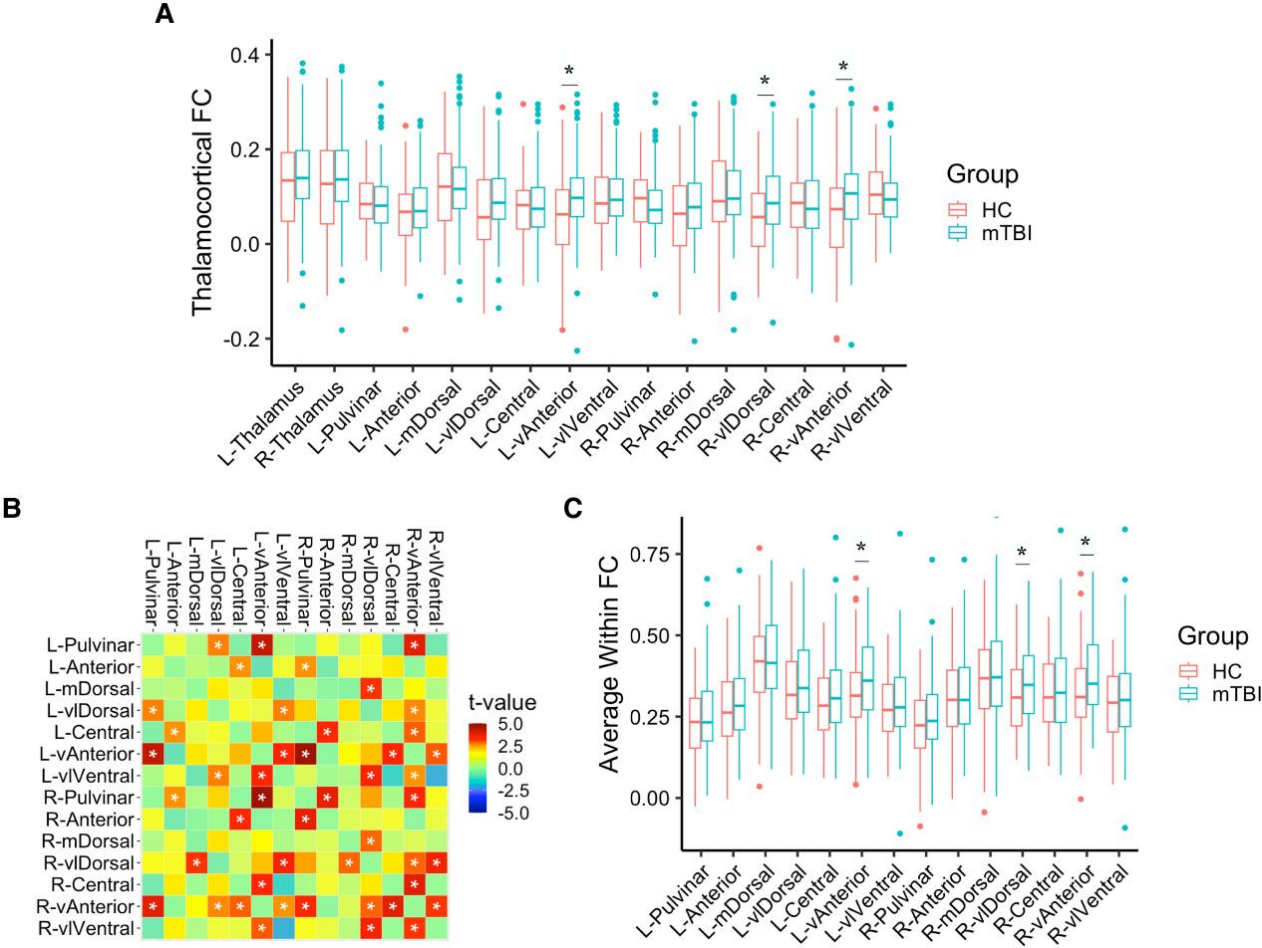
**Nuclei-specific vulnerability comparing mTBI and controls.** Asterisk indicates statistical significance at FDR-corrected *P* ≤ 0.05, HC = controls. (**A**) Thalamocortical connectivity comparisons. (**B**) Within-thalamus connectivity adjacency matrix by *t*-value colour from statistical testing, where red-yellow colours indicate higher functional connectivity in mTBI compared to controls. (**C**) Average within-thalamus connectivity values, derived from **B**, showing higher functional connectivity in mTBI in the same three nuclei as in **A**.

Several lines of evidence suggest widespread functional alterations in acute mTBI, despite no differences in thalamic volume (Table 2). First, average global functional connectivity between the thalamic ROIs and cortical grey matter were significantly different between the two groups, with significant nuclei-specific global hyperconnectivity from the bilateral ventral anterior [vAnterior; Left *F*(1,180) = 10.5, *P* = 0.02; Right *F*(1,180) = 8.34, *P* = 0.02] and right ventral lateral dorsal [vlDorsal; *F*(1,180) = 9,89, *P* = 0.02] in patients compared to controls after FDR correction (Fig. 1A and Table 2). We found further evidence for vulnerability of these specific nuclei when considering within-thalamus connectivity. Across 23 pairs of nuclei, patients showed increased connectivity compared to controls (Fig. 1B), and additionally the averaged connectivity to the rest of the thalamus for the left and right vAnterior and the right vlDorsal thalamic nuclei was significantly higher in patients versus controls (Table 2 and Fig. 1C). No decreases in within-thalamus functional connectivity were found in mTBI.

**Table 2:** Mild TBI versus controls comparisons in structural and functional imaging

Thalamic ROI	T_1_-weighted volume *F*-test (1,179)	Thalamocortical FC *F*-test (1,180)	Average within-thalamus FC *F*-test (1,180)
Left Thalamus	*F* = 0.9, *P* = 0.96	*F* = 4.1, *P* = 0.10	–
Right Thalamus	*F* = 0.02, *P* = 0.97	*F* = 2.7, *P* = 0.20	–
**Left-hemisphere nuclei**			
Pulvinar	*F* = 0.1, *P* = 0.97	*F <* 0.*01*, *P* = 0.99	*F* = 2.0, *P* = 0.24
Anterior	*F* = 3.7, *P* = 0.32	*F* = 4.7, *P* = 0.08	*F* = 1.5, *P* = 0.25
mDorsal	*F* = 0.05, *P* = 0.97	*F* = 0.7, *P* = 0.61	*F* = 1.5, *P* = 0.25
vlDorsal	*F* = 0.4, *P* = 0.97	*F* = 6.0, *P* = 0.06	*F* = 4.8, *P* = 0.08
Central	*F <* 0.*01*, *P* = 0.99	*F* = 0.4, *P* = 0.63	*F* = 2.6, *P* = 0.24
**vAnterior**	*F* = 1.7, *P* = 0.80	***F* = 10.5, *P* = 0.02**	***F* = 7.8, *P* = 0.03**
vlVentral	*F* = 0.01, *P* = 0.97	*F* = 1.4, *P* = 0.41	*F* = 1.4, *P* = 0.26
**Right-hemisphere nuclei**			
Pulvinar	*F* = 4.5, *P* = 0.31	*F* = 1.3, *P* = 0.41	*F* = 5.2, *P* = 0.08
Anterior	*F* = 6.6, *P* = 0.18	*F* = 5.7, *P* = 0.06	*F* = 1.1, *P* = 0.30
mDorsal	*F* = 0.1, *P* = 0.97	*F* = 0.4, *P* = 0.64	*F* = 1.8, *P* = 0.24
**vlDorsal**	*F* = 0.1, *P* = 0.97	***F* = 9.9, *P* = 0.02**	***F* = 9.0, *P* = 0.02**
Central	*F* = 0.6, *P* = 0.97	*F* = 0.05, *P* = 0.87	*F* = 1.9, *P* = 0.24
**vAnterior**	*F* = 1.2, *P* = 0.90	***F* = 8.3, *P* = 0.02**	***F* = 9.0, *P* = 0.02**
vlVentral	*F* = 0.1, *P* = 0.97	*F* = 0.3, *P* = 0.65	*F* = 2.3, *P* = 0.24

Bold indicates statistical significance at FDR-corrected *P* ≤ 0.05, whereby tests are two-tailed but patients showed increased functional connectivity compared to controls in significant results. Tests included covariates of sex and age, and additionally spatial normalization quality for volume comparisons. FC = functional connectivity.

**Figure 2:**
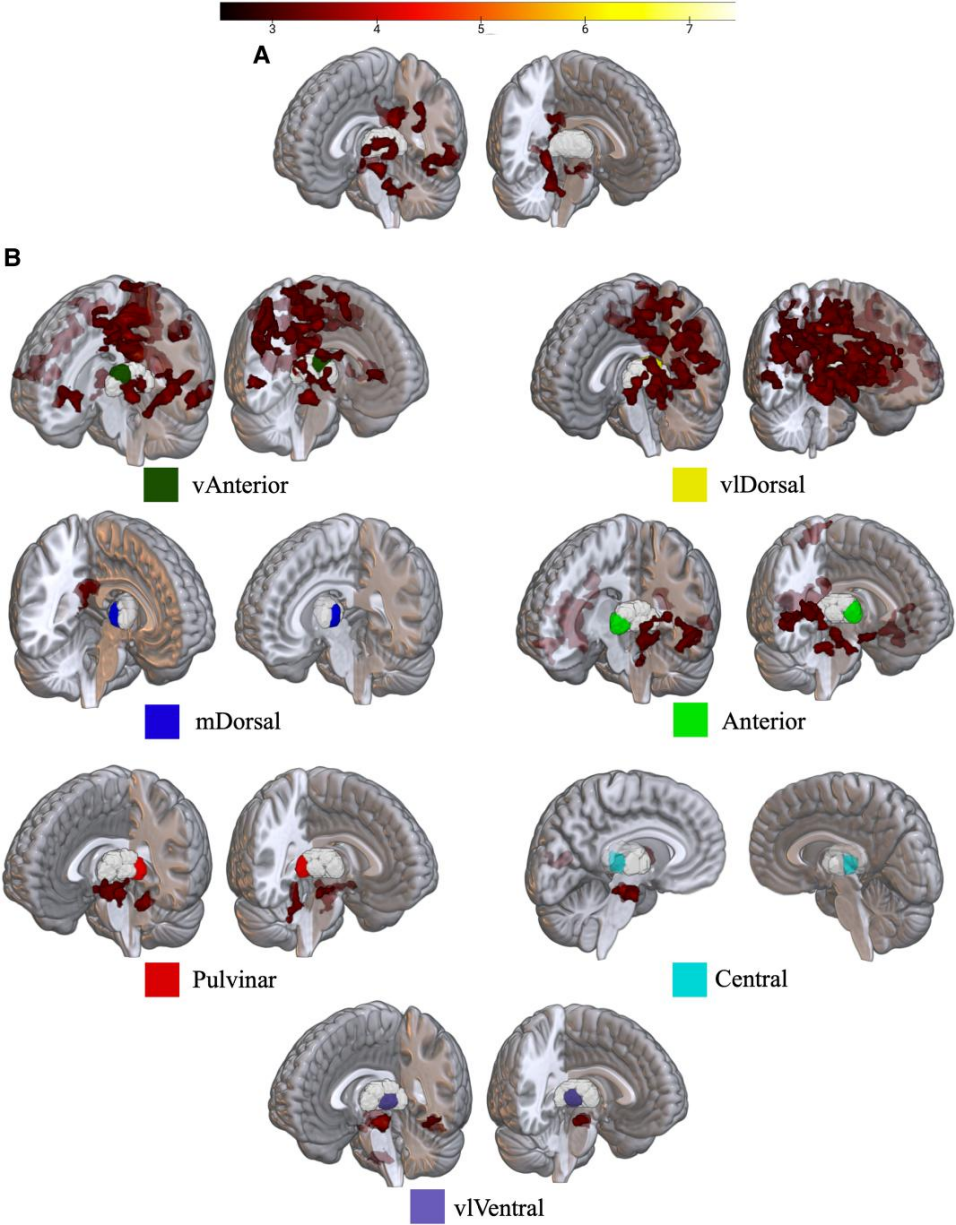
**Voxel-wise results of increased functional connectivity in mTBI compared to controls.** All images show voxels surviving significance and cluster-level correction, using colour-bar scale at top. (**A**) Results from left and right thalamus respectively, where seed mask is presented in greyscale. (**B**) Left and right hemisphere nuclei-specific results, where seed-nucleus is indicated by the colour legend. Images without clusters shown indicate no voxels exceeded cluster-corrected significance. *Top left*: Results seeded from vAnterior nuclei; *top right*: results from vlDorsal nuclei, partially obscured by hyperconnected clusters.

We next looked for specific connectivity changes that underpinned the globally increased thalamocortical connectivity. The mTBI patients showed increased functional connectivity from all thalamic ROIs, except for the right-Central and right-mDorsal nuclei. In contrast, no ROI demonstrated a decrease in connectivity. This picture of acute hyperconnectivity could be split into three groups of nuclei-specific results (Fig. 2); at posterior cingulate cortex from more anterior thalamic nuclei (Anterior, vAnterior, mDorsal and vlDorsal); midbrain region inferior to the left red nucleus (max coord: −4, −22, −17) from more posterior thalamic nuclei (Pulvinar, Central, vlVentral); and widespread cortical hyperconnectivity from vAnterior and vlDorsal nuclei, replicating the results of global increases in thalamocortical connectivity. These results are echoed in overarching voxel-wise results from the left and right thalamus (Fig. 2A), but greater specificity was found by looking at the respective subdivisions (Fig. 2B).

### Common markers of injury do not differentiate chronic outcomes

Blood biomarker levels were available for an average of 84 participants per marker (range 79–92) and collected at a mean of 2.98 (SD 6.80) days post-injury. These showed no significant differences between groups in NSE [*F*(1,86) = 1.74, *P* = 0.38; *F*(1,82) = 0.07, *P* = 0.79], S100B [*F*(1,87) = 4.08, *P* = 0.28; *F*(1,83) = 1.35, *P* = 0.58], GFAP [*F*(1,76)= 1.33, *P* = 0.38; *F*(1,72) = 0.76, *P* = 0.58], Tau [*F*(1,76) = 0.02, *P* = 0.89; *F*(1,72) = 0.19, *P* = 0.79], UCH-L1 [*F*(1,72) = 0.76, *P* = 0.46; *F*(1,68) = 1.00, *P* = 0.58] or NFL [*F*(1,75) = 1.56, *P* = 0.38; *F*(1,71) = 1.50, *P* = 0.58] between GOSE or PCS groups, respectively. The absence of visible structural damage, neuropsychiatric disease, previous concussion or these blood biomarkers in our poor outcome groups underlined the need for novel biomarkers to help prognosticate chronic outcome.

### Acute thalamic hyperconnectivity is related to chronic post-concussive symptoms

**Figure 3:**
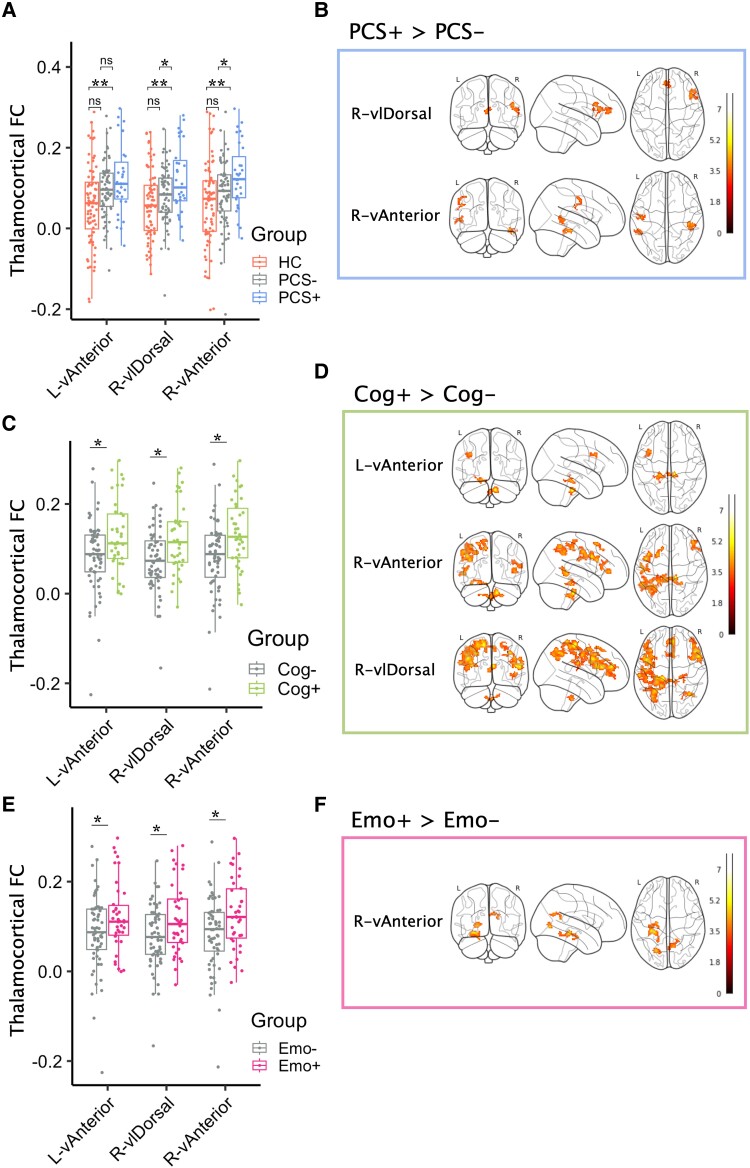
**Relating thalamic hyperconnectivity to post-concussive outcomes.** (**A, C** and **E**) Comparison of average thalamocortical functional connectivity between outcome groups looking at the three nuclei of interest; left and right vAnterior and right vlDorsal. Asterisk indicates statistical significance at *P* ≤ 0.05. (**B, D** and **F**) Column shows voxel-wise thalamic functional connectivity results seeded from these same nuclei surviving significance and cluster-level correction, compared between corresponding outcome groups. These results show higher functional connectivity in those with PCS, and cognitive/emotional symptom clusters, at the local and global level of investigation.

Group comparisons showed greater thalamic functional connectivity at both local and global levels in patients with chronic post-concussive symptoms (PCS+) than those without such symptoms (PCS−) (Table 3 and Fig. 3A). The PCS+ group showed clusters of increased connectivity between R-vAnterior and middle/inferior temporal gyrus, and R-vlDorsal and inferior frontal gyrus and frontal cingulate/paracingulate (Fig. 3B). No connectivity differences were seen between patients with complete or incomplete recovery based on the GOSE. Furthermore, thalamocortical connectivity was higher in those with cognitive or emotional symptoms from all three nuclei (Table 3 and Fig. 3C and E). Somatic symptoms were associated with significant but less prominent cortical hyperconnectivity from the right vAnterior nucleus (Table 3). Hyperconnected clusters in those with cognitive symptoms mainly encompassed cortical regions associated with frontoparietal control network, with some additional increased connectivity to midbrain regions (Fig. 3D). Participants with long-term emotional symptoms also displayed hyperconnectivity seeded from the right vAnterior nucleus to medial temporal and medial posterior occipital regions, which have previously been associated with emotion/language and visual networks (Fig. 3F). Regional network relationships were identified using the ICN-Atlas toolbox in SPM,^54^ described in Supplementary Fig. 5. No voxel-wise differences were found associated with somatic symptom presentation. However fewer individuals presented somatic symptoms on average, and as such had more unequal sample sizes, which may have reduced statistical sensitivity to find an effect. No differences were found between outcome groups in within-thalamus functional connectivity comparisons (Supplementary Table 2).

**Table 3:** Outcome group comparisons in thalamocortical functional connectivity

Comparison	Test (df)	Acute thalamocortical functional connectivity		
		Left vAnterior	Right vAnterior	Right vlDorsal
**6-Month GOSE**				
GOSE≤7 > Control	*F*-test (1,120)	***F* = 7.3, *P* = 0.02**	***F* = 8.1, *P* = 0.02**	***F* = 11.1, *P* = 0.01**
GOSE-8 > Control	*F*-test (1,130)	***F* = 6.8, *P* = 0.02**	*F* = 3.8, *P* = 0.08	*F* = 4.5, *P* = 0.06
GOSE≤7 > GOSE-8	*F*-test (1,100)	*F* = 0.01, *P* = 0.91	*F* = 0.9, *P* = 0.38	*F* = 1.8, *P* = 0.23
**6-Month PCS**				
PCS+ > Control	*F*-test (1,103)	***F* = 9.5, *P* = 0.01**	***F* = 10.7, *P* = 0.01**	***F* = 13.2, *P* = 0.004**
PCS− > Control	*F*-test (1,139)	*F* = 4.3, *P* = 0.06	*F* = 2.2, *P* = 0.14	*F* = 2.9, *P* = 0.12
PCS+ > PCS−	*F*-test (1,92)	*F* = 2.3, *P* = 0.14	*F* = 5.0, *P* = 0.050*	***F* = 5.8, *P* = 0.04**
**6-month Rivermead factor structure**				
Cog+ > Cog−	*F*-test (1,92)	***F* = 6.7, *P* = 0.02**	***F* = 9.7, *P* = 0.01**	***F* = 9.7, *P* = 0.01**
Emo+ > Emo−	*F*-test (1,92)	*F* = 4.3, *P* = 0.052*	***F* = 6.5, *P* = 0.02**	***F* = 6.6, *P* = 0.02**
Som+ > Som−	*F*-test (1,92)	*F* = 2.5, *P* = 0.12	***F* = 5.1, *P* = 0.04**	*F* = 3.4, *P* = 0.08

Bold indicates statistical significance at FDR-corrected *P* ≤ 0.05. *F*-test statistics are derived from linear models comparing between-groups after accounting for covariates, equivalent to between-subjects *t*-test. Tests were two-tailed, however the poorer outcome group (i.e. GOSE ≤7 or PCS/symptom positive), or mTBI group compared to controls, had higher functional connectiivty. *Values rounded to *P* = 0.05. df = degrees of freedom.

### Neurochemical associations of hyperconnectivity may identify treatment targets

**Figure 4:**
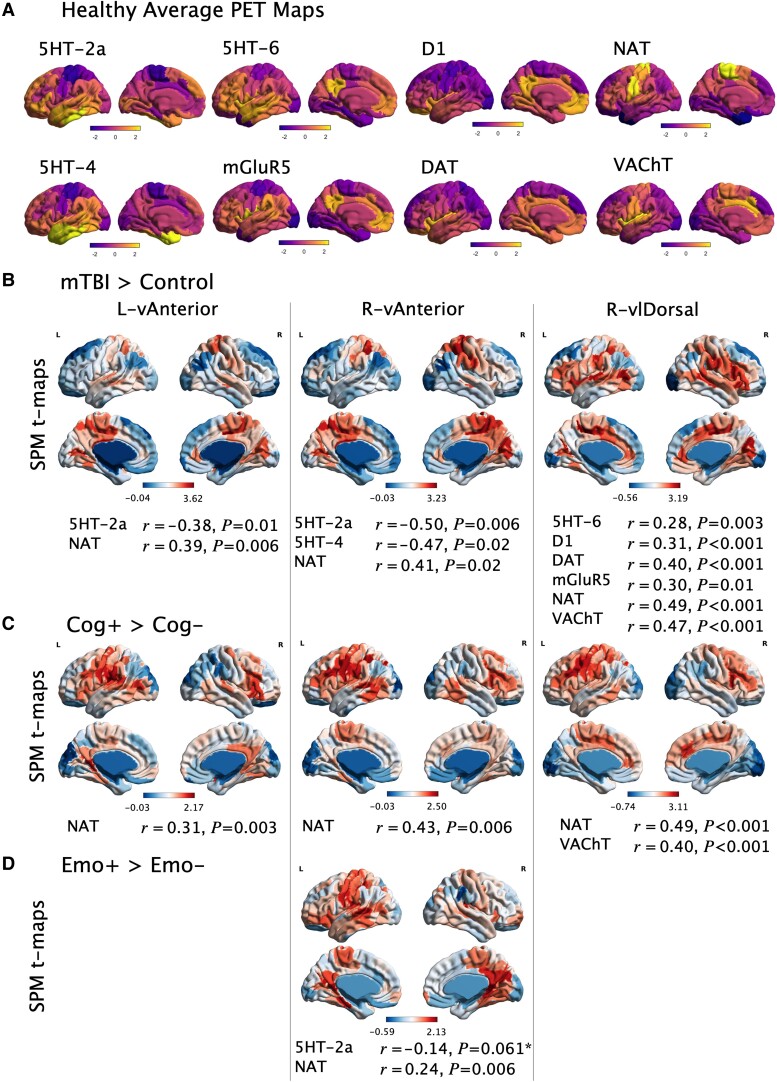
**Significant correlations between averaged PET maps and voxel-wise SPM-*t* maps from group comparisons. (A**) PET maps reaching significant association in one or more comparison, each normalized within-map to show range of *z*-scores. Higher *z*-score indicates greater density of that transmitter receptor or transporter. (**B**–**D**) Cortical SPM *t*-maps derived from groups comparisons of functional connectivity seeded from each respective thalamic ROI, where red regions indicate greater connectivity in one group (mTBI, Cog+, Emo+) than the second group (Control, Cog−, Emo−). These *t*-maps are correlated with PET maps and significant associations presented below. *Marginally non-significant when using Schaefer200 parcellation but found to be significant when using alternative parcellations (Glasser360 and Schaefer100).

In relating regions of injury-induced thalamic connectivity to neurotransmitter maps, regions rich in monoaminergic transmitter receptors and transporters were targets of thalamic hyperconnectivity after mTBI—with positive correlations to noradrenergic and dopaminergic targets, and negative correlations to select serotonergic transmitter system constituents. Positive associations were also found for metabotropic glutamate and vesicular acetylcholine targets. Most strikingly, a significant positive correlation between hyperconnectivity and noradrenergic transporter density was found across all three nuclei of interest for the mTBI-Control and Cog+/Cog− comparisons, and the investigated Emo+/Emo− *t*-map. This suggests that regions which are functionally hyperconnected after injury and associated with persistent specific symptomatology have high noradrenergic transporter density. A similar relationship was also found for lower 5-HT 2a receptor levels and Emo+/Emo− *t*-maps; although this latter result marginally exceeded the significance threshold in one of the tested parcellations (Glasser360 *P* = 0.007; Schaefer100 *P* = 0.033; Schaefer200, *P* = 0.064). Results that remained significant after stringent statistical corrections are presented in Fig. 4. These are derived from Schaefer's local-global parcellation with 200 cortical regions^41^ but are only shown if also replicated as significant using the 100-region Schafer parcellation (Schaefer-100), as well as Glasser's well-known multimodal parcellation (360 cortical regions).^42^ The remaining nonsignificant associations with PET maps are presented in Supplementary Table 3.

### Longitudinal evolution of thalamic connectivity varies with post-concussive outcome

Finally, given the potential prognostic value of thalamocortical functional conectivity, we investigated a subset of patients (*n* = 31) in whom structural and functional imaging were available at 6 and 12 months post-injury. The serial imaging cohort did not differ in age [*X*^2^(1) = 0.8, *P* = 0.69] or baseline GCS (Fisher's exact, *P* = 0.59) to the cohort in whom serial imaging was unavailable, but had higher incomplete recovery according to GOSE at 6 and 12 months [*X*^2^(1) = 15.3, *P* < 0.001; *X*^2^(1) = 15.3, *P* < 0.001], fewer female participants [*X*^2^(1) = 4.0, *P* = 0.046], and were exclusively from admission stratum due to recruitment protocols in CENTER-TBI [*X*^2^(1) = 36.4, *P* < 0.001]. Furthermore, *n* = 16 of this cohort developed PCS at either/both 6/12 months versus *n* = 15 who did not, encompassing poorer outcomes than the original cohort. This cohort therefore provides a representation of real-world follow up practice and may be less generalizable to mTBI as a whole. However, the serial imaging cohort showed acute thalamocortical hyperconnectivity compared to controls in the same three nuclei as seen in the overall mTBI group after FDR-correction [L-vAnterior *F*(1,104) = 8.46, *P* = 0.03; R-vAnterior *F*(1,104) = 7.45, *P* = 0.04; R-vlDorsal *F*(1,104) = 11.96, *P* = 0.01] and were therefore thought to be appropriately representative of the overall narrative of pathophysiology in mTBI.

**Figure 5:**
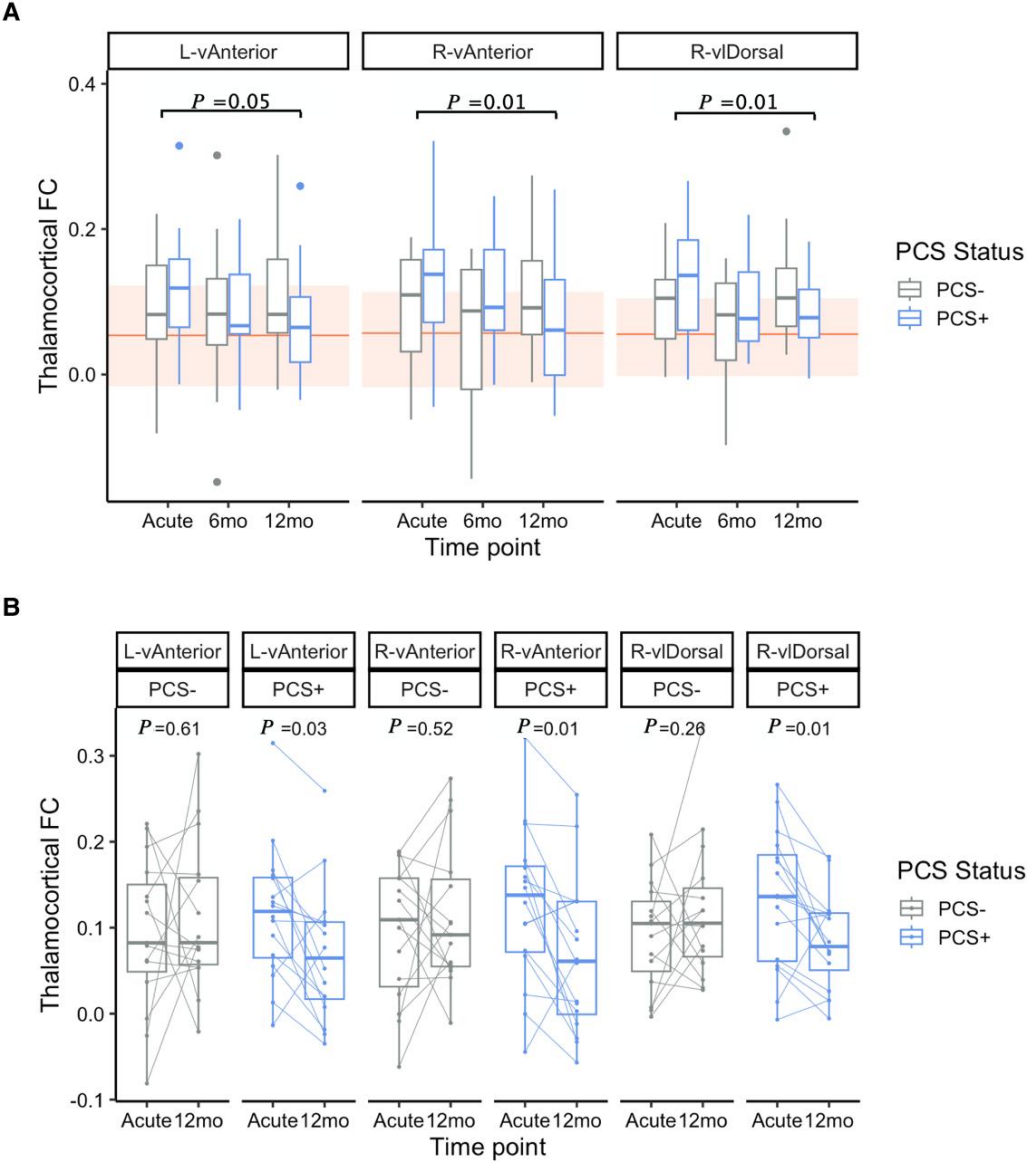
**Longitudinal follow-up of thalamocortical functional connectivity in three nuclei of interest in relationship to PCS.** (**A**) Mixed ANOVA between acute and 12-month time-points between groups, where *P*-values given are significant interaction effects between time-point (acute or 12-month) and group (PCS+ or PCS−). Shaded regions give the IQR of controls for each nucleus, with solid line indicating the controls’ mean. (**B**) *Post hoc* results within-subjects finding significant decreases in functional connectivity only in those with PCS. Lines join individual subjects’ data at different time-points. All *P*-values in **A** and **B** are uncorrected for multiple comparisons due to small sample size, however corrected values are presented in-text.

When splitting this serial imaging cohort into PCS+ and PCS−, all three nuclei of interest showed significant interaction effects between PCS status and time (acute and 12-month imaging) using a two-way mixed ANOVA [L-vAnterior *F*(1,24) = 4.42, *P* = 0.046; R-vAnterior *F*(1,24) = 8.11, *P* = 0.01; R-vlDorsal *F*(1,24) = 7.94, *P* = 0.01]. *Post hoc*, within-subjects, tests showed that only the PCS+ cohort showed significantly decreased functional connectivity in these nuclei over time [L-vAnterior *F*(1,11) = 6.18, *P* = 0.03; R-vAnterior *F*(1,11) = 8.42, *P* = 0.01; R-vlDorsal *F*(1,11) = 10.1, *P* = 0.01], whereas the PCS- cohort showed no change [L-vAnterior *F*(1,9) = 0.28, *P* = 0.61; R-vAnterior *F*(1,9) = 0.45, *P* = 0.52; R-vlDorsal *F*(1,9) = 1.46, *P* = 0.26]. These results can be seen in Fig. 5 and are uncorrected for multiple comparisons, given the small sample sizes in this follow-up cohort. Whilst these were not explicitly compared to control groups, Fig. 5 shows the healthy control mean and interquartile range to provide additional context. These results were reproduced when analysing only significantly hyperconnected clusters in mTBI compared to controls derived from voxel-wise maps: functional connectivity in initially hyperconnected clusters decreases over time only in those with long-term PCS (Supplementary Fig. 6). Additionally, volume analyses were repeated at these time-points and showed no changes to controls, or within mTBI over time (Supplementary Table 4). This suggests that time-dependent functional imaging changes associated with poor outcome are not reflected in routine structural imaging.

## Discussion

Persistent symptoms and incomplete recovery are common following mTBI, but the neural substrates of these poor outcomes are unclear; limiting our ability to prognosticate outcome or identify therapeutic targets. Our results confirm that recovery from mTBI is functionally and/or symptomatically incomplete in almost half of mTBI participants at 6 months post-injury. We show that ‘mild’ injury is associated with widespread increases in acute connectivity of thalamic nuclei; to cortical, subcortical, and other thalamic regions. This is in the absence of detectable structural change. Further, these changes are uniquely associated with the presence of persistent post-concussive symptoms, and not general functional outcome, with specific relationships identifiable between individual thalamic nuclei and symptom categories. Finally, injury-induced connectivity changes show relationship to monoaminergic neurochemical profiles in target cortical regions and evolve differentially in mTBI patients in whom symptoms persist over time. The absence of key markers of poor outcome (CT abnormalities, past neurological or psychiatric disease, or previous concussion)^3,55^ in our cohorts implies that acute functional change and chronic symptomatology are found in even the ‘mildest’ form of mTBI.

We thus propose that acute thalamic functional connectivity has prognostic potential for enduring post-concussive symptoms, with particular importance of the vAnterior and vlDorsal nuclei groups. Crucially, functional imaging may provide earlier markers for poor outcome than routine anatomical imaging, as behaviourally-relevant structural thalamic alterations have been previously found in ‘post-acute’ mTBI^15,16^ but were not found here in the ‘acute’ investigation. Additionally, we did not find association of acute blood biomarkers of injury to chronic outcome, nor thalamic structural change over time beyond this acute phase. These findings suggest our ‘mildest’ mTBI is a functionally-driven disorder which requires functional markers of outcome.

Previous literature has found potential resting-state biomarkers of outcome in alterations of network connectivity.^56,57^ Whilst this vast literature has some mixed findings,^58,59^ it nevertheless suggests a global-scale of alterations,^60^ which may relate to our global thalamic connectivity changes in mTBI. Indeed, emerging evidence shows thalamic nuclei are capable of multimodal integration across cortical networks, and thalamic injury in severe TBI induces widespread changes in network dynamics, proposing the thalamus as a critical integrative hub which can influence cortical dynamics.^5,61^ We therefore suggest the thalamus warrants greater investigation, to better understand injury-induced alterations and how these might influence existing network-level markers of outcome, by studying the thalamus and network function in tandem. Moreover, a heterogenous and global disorder such as mTBI may be better understood by investigating a globally-relevant hub such as the thalamus. Supporting this, we find acute thalamic hyperconnectivity from vAnterior and vlDorsal nuclei in mTBI, specifically associated with chronic post-concussive symptoms. Our results converge with previous findings of thalamic hyperconnectivity in small samples,^20–23^ and provide a potential early marker of symptom-specific chronic outcome.

Thalamic hyper- as opposed to hypo- connectivity is consistent across all avenues of investigation and decreases over time are only found in those with persistent symptoms. Hyperconnectivity is an increasingly common signature of acute injury^53,62^ and may indicate specific neuronal damage^21^ leading to less signal variability and thus increased ‘connectivity’ or perhaps an adaptive response aiming to overcome such injury. Indeed, several studies of moderate and severe TBI have directly tested and support this adaptive hyperconnectivity hypothesis, proposing it as a compensatory response.^63–65^ However, the mild TBI literature faces greater speculation on what is adaptive or maladaptive.^58^ Further work in mTBI^53^ and other neurodegenerative disorders^66^ posits a time-dependent change from acute hyper- to chronic hypo- connectivity as potentially adaptive mechanisms fatigue from persistent overstimulation, particularly in those with poor outcomes, whereas successful recovery is characterized by long-term recovery of connectivity to healthy levels.^53^ Here, we find preliminary evidence for decreasing connectivity into healthy ranges, with significantly decreased thalamocortical connectivity only in those with chronic symptoms, partially supporting previously proposed models.^53^ Such relationships were additionally found in highly connected ‘hub’ regions from voxel-wise investigations, specifically affecting the posterior cingulate and the insular cortices. These hubs have been previously identified to be relevant in mTBI thalamic connectivity,^21^ and are also more affected in other neurological diseases such as Alzheimer's and Parkinson's.^66^ These may be particularly vulnerable as damaged nodes lower in the connectomic hierarchy offload to higher-level hubs,^66^ leading to acute hyperconnectivity, particularly in these regions. Thus, our findings may suggest a fatigue of initially adaptive hyperconnectivity mechanisms in those with poor outcome, particularly affecting connectivity hubs. This requires greater investigation to establish the underlying physiology of an adaptive response to injury in mTBI and its causality in outcome.

We further explored therapeutic targets of our potential prognostic markers and found that thalamic functional connectivity was associated in symptom-specific fashion with particular neurotransmitter system profiles converged on the importance of monoaminergic transmitter systems. More specifically, the analysis showed associations of hyperconnectivity with noradrenaline transporter and 5HT-2a receptor for cognitive and emotional symptoms, respectively. These powerful neuromodulatory systems^67^ are central to the maintenance of healthy connectivity profiles in the human brain.^68,69^ In the context of this cohort, it is plausible that noradrenergic and serotonergic (or broader monoaminergic) systems are involved in producing the input-output relationships required for compensatory hyperconnectivity, which is affected when these systems become/remain dysfunctional. Consequently, our data suggest that transmitter system changes might also operate in mTBI—not just in severe cases as previously suggested,^39^ and that these relationships represent biomarkers that have therapeutic specificity. Expressly, individuals who show noradrenaline-associated connectivity alterations might respond to drugs such as methylphenidate.^70^ Similarly, the relevance of a serotonergic target for emotional symptoms after injury is intuitive in the context of pre-existing TBI and depression literature^39^ and thus might represent a domain-specific therapeutic direction for future investigations. Therein, these non-invasive, easily-implementable assessments could allow for precision neurotransmitter/neuromodulator therapeutic strategies to be developed in the context of mTBI.

With patient care in mind, the particular relevance of vAnterior and vlDorsal nuclei to injury and outcome is interesting to consider. Their specific involvement may be related to their highly GABA-ergic innervation;^71^ which represents ∼35% of total neuronal populations in the vAnterior nucleus.^72^ The vAnterior forms part of the thalamic motor relay alongside the ventrolateral nucleus, connecting GABA-rich substantia nigra pathways up to the premotor cortex, whereas vlDorsal nuclei project to the posterior cingulate.^73^ There are further efferent projections from vAnterior thalamus to primary motor, supplemental motor, and possible prefrontal regions, suggesting vAnterior is important for long-range cortical modulatory loops. A previous study investigating ventrally-defined thalamic nuclei overlapping our nuclei indeed found both increased thalamocortical connectivity in acute mTBI and increased indicators of neuronal loss and dysfunction using magnetic resonance spectroscopy.^21^ The authors speculated that these findings could be due to loss of thalamic inhibitory GABAergic interneurons reducing inhibitory control.

Indeed, excitatory-inhibitory imbalance is a known consequence of TBI^74^ and has shown links to thalamocortical functional connectivity regulation^75^ and fMRI-derived resting-state networks with strongest association to concurrent GABA-A binding potential.^76^ GABA-related changes are also found in animal models of TBI, showing downregulation of GABA-A and GABA-B receptor subunit mRNAs related to thalamocortical relay degeneration^77^ and chronically-reduced GABAergic parvalbumin positive interneurons.^78^ Whilst we did not find a specific association between acute functional connectivity and GABA-A in PET correlations, we only investigated cortical, rather than subcortical thalamic GABA-A binding, a limitation given that only the thalamus, and not its functionally hyperconnected regions (e.g. posterior cingulate), showed these markers of neuronal loss.^21^ Furthermore, given the well-defined association between TBI and GABAergic parvalbumin positive interneurons,^74^ it may be that such associations are clouded by the lack of neurochemical subtype specificity of GABA-A PET maps. We therefore speculate that the present results of ventral thalamic hyperconnectivity replicated across different measures may be associated with thalamic GABA-related inhibitory imbalance, which warrants further investigation.

There are arguably four main limitations of this study. First, the thalamus and its subdivisions were not individually defined in each patient. While previous work has validated atlas suitability and accuracy when individual parcellation is not possible,^32^ individual parcellation could provide higher anatomical accuracy. There is a lack of consensus on thalamic subdivisions’ terminology^79^ or a widely accepted thalamic atlas for imaging studies,^80^ which should be considered when comparing the present results to other studies. When considering our thalamus-derived measures, multicentre harmonization development is ongoing in the neuroimaging community to better deal with confound of multi-site data collection. The present method has previously been shown to robustly account for multisite differences across imaging modalities including structural and functional imaging but is one of many possible techniques. Using such methods should be done with caution as they could alter imaging-derived phenotypes inappropriately if not used correctly, as highlighted by Richter *et al*.^46^ Secondly, prevalence rates of PCS in mTBI populations vary substantially depending on the classification method used, however the most common method in the literature aligns with ICD-10 criteria as used here.^81^ There are further discrepancies in what constitutes an ‘experienced’ symptom. As in many previous studies, we used a less conservative definition and thus may incur some ‘falsely’ defined mTBI patients with PCS.^82^ We additionally highlight that our cognitive and emotional groups showed overlap. Whilst post-concussive symptoms may cluster in this three-factor structure, some authors have suggested alternative symptom domains,^83^ and indeed individuals can concurrently present any number of symptoms. Future research should investigate cohorts uniquely presenting these symptoms for more targeted therapeutic outputs. Thirdly, blood-based biomarker levels can vary substantially over time post-injury. Data available for this cohort had large variation in mean time since injury, and we aimed to keep sample sizes as large as possible by their inclusion. We therefore cannot exclude the possibility that acute blood-biomarker levels had early changes from control levels, which were not detectable in our data. Finally, we aimed to obtain hypothesis-setting results regarding the neurochemical associations of thalamic hyperconnectivity. However, correlating functional connectivity maps from clinical populations to averaged healthy neurochemical profiles is only the first step in this direction. Neurotransmitter systems are globally disturbed after injury and may not be best represented by these average healthy PET maps. Our analysis only addressed cortical relationships, a shortcoming given that we additionally found subcortical clusters of connectivity change. Further, only a subset of all possible neurotransmitters was available for investigation, such that other non-investigated neurochemical profiles may be important. Nevertheless, this recently-developed method encompasses the broadest set of *in vivo* neurotransmitter maps available to date for the human brain, and it begins to investigate biological systems within statistical frameworks of neuroimaging research, bridging fields with traditionally little communication, an important step in imaging-guided treatment.

The ‘mild’ TBI population is growing and is insufficiently supported. Our results show that acute thalamic connectivity may provide an avenue to better understand, prognosticate and potentially guide treatment of chronic post-concussive symptoms after mTBI. The explicit predictive power of these measures should firstly be assessed in an independent sample, with focus on the longitudinal evolution of connectivity and its relationship to recovery. Indeed, longitudinal studies such as ours are limited and hold great power to influence clinical practice and long-term care plans, as we find symptom-relevant neurological change extends well-beyond 6 months. Future work should aim to integrate multimodal microstructural imaging within clinical populations to better understand the links between acute injury and increased functional connectivity, such as links between thalamic connectivity and existing network-level biomarkers, GABA-related effects, and development of monoaminergic drug treatments. It will be important that these latter findings are further developed through assessments of blood/salivary biomarkers of neurotransmitter metabolites, and whether integrity and/or connectivity of the brainstem sources of these transmitters to the thalamus and the rest of the brain are perturbed.^84,85^ These steps will advance our understanding of mTBI across multiple scales of investigation to promote more informed predictive models and patient care, whereby thalamic alterations may be a key component in this direction.

## Supplementary Material

awad056_Supplementary_Data

## Appendix 1

### CENTER-TBI MRI substudy participants and investigators

Krisztina Amrein, Nada Andelic, Lasse Andreassen, Audny Anke, Philippe Azouvi, Bo-Michael Bellander, Habib Benali, Andras Buki, Alessio Caccioppola, Emiliana Calappi, Marco Carbonara, Giuseppe Citerio, Hans Clusmann, Mark Coburn, Jonathan Coles, Marta Correia, Endre Czeiter, Véronique De Keyser, Vincent Degos, Bart Depreitere, Live Eikenes, Erzsébet Ezer, Kelly Foks, Shirin Frisvold, Alexandre Ghuysen, Damien Galanaud, Ben Glocker, Asta Haberg, Iain Haitsma, Eirik Helseth, Peter J. Hutchinson, Evgenios Kornaropoulos, Noémi Kovács, Ana Kowark, Steven Laureys, Didier Ledoux, Hester Lingsma, Andrew I. R. Maas, Geoffrey Manley, David K. Menon, Tomas Menovsky, Benoit Misset, Visakh Muraleedharan, Ingeborg Nakken, Virginia Newcombe, Wibeke Nordhøy, József Nyirádi, Fabrizio Ortolano, Paul M. Parizel, Vincent Perlbarg, Paolo Persona, Wilco Peul, Jussi P. Posti, Louis Puybasset, Sophie Richter, Cecilie Roe, Olav Roise, Rolf Rossaint, Sandra Rossi, Daniel Rueckert, Toril Skandsen, Abayomi Sorinola, Emmanuel Stamatakis, Ewout W. Steyerberg, Nino Stocchetti, Riikka Takala, Viktória Tamás, Olli Tenovuo, Zoltán Vámos, Gregory Van der Steen, Wim Van Hecke, Thijs Vande Vyvere, Jan Verheyden, Anne Vik, Victor Volovici, Lars T. Westlye, Guy Williams, Stefan Winzeck, Peter Ylén, and Tommaso Zoerle.
