# Supplementary material for: Acute thalamic connectivity precedes chronic post-concussive symptoms in mild traumatic brain injury
Source: Brain. 2023 Feb 22;146(8):3484–99. doi: 10.1093/brain/awad056 (PMC10393415; doi:10.1093/brain/awad056)

## SUPPLEMENTARY MATERIALS

**Fig S1. Consort diagram for patient inclusion.** All data were obtained from CENTER-TBI CORE v3.0.

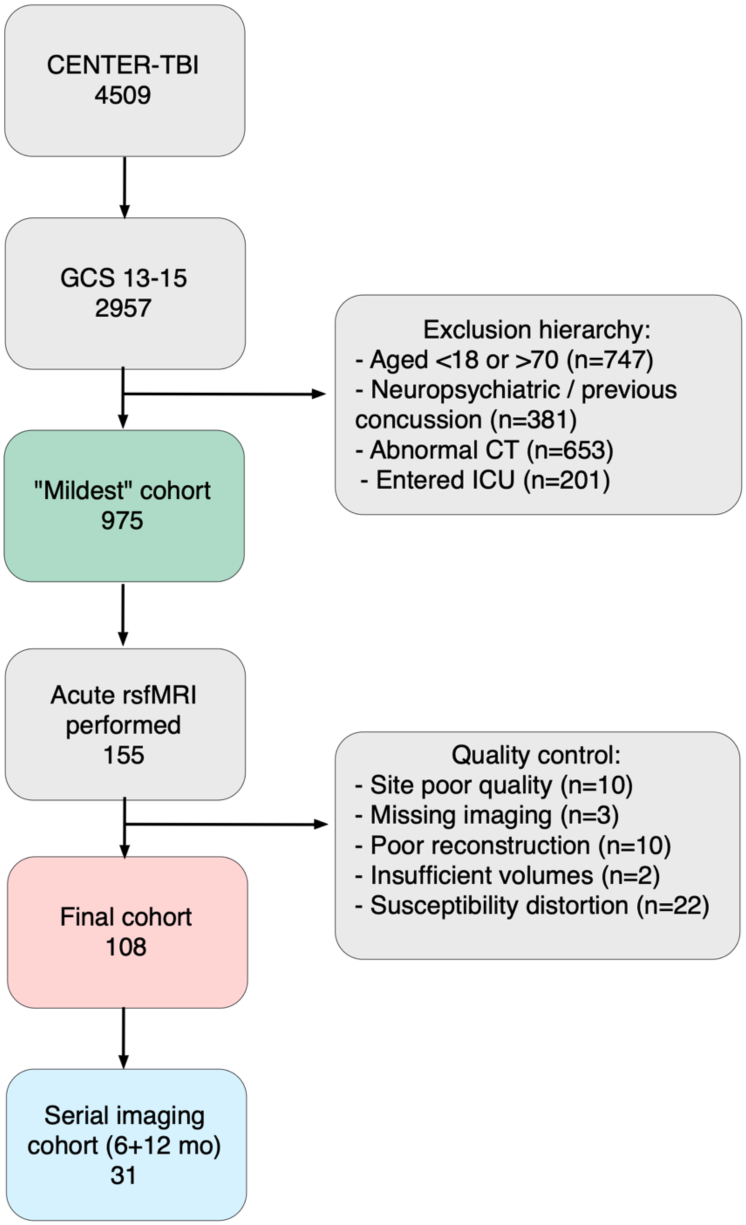

**Fig S2. Methods Overview.**

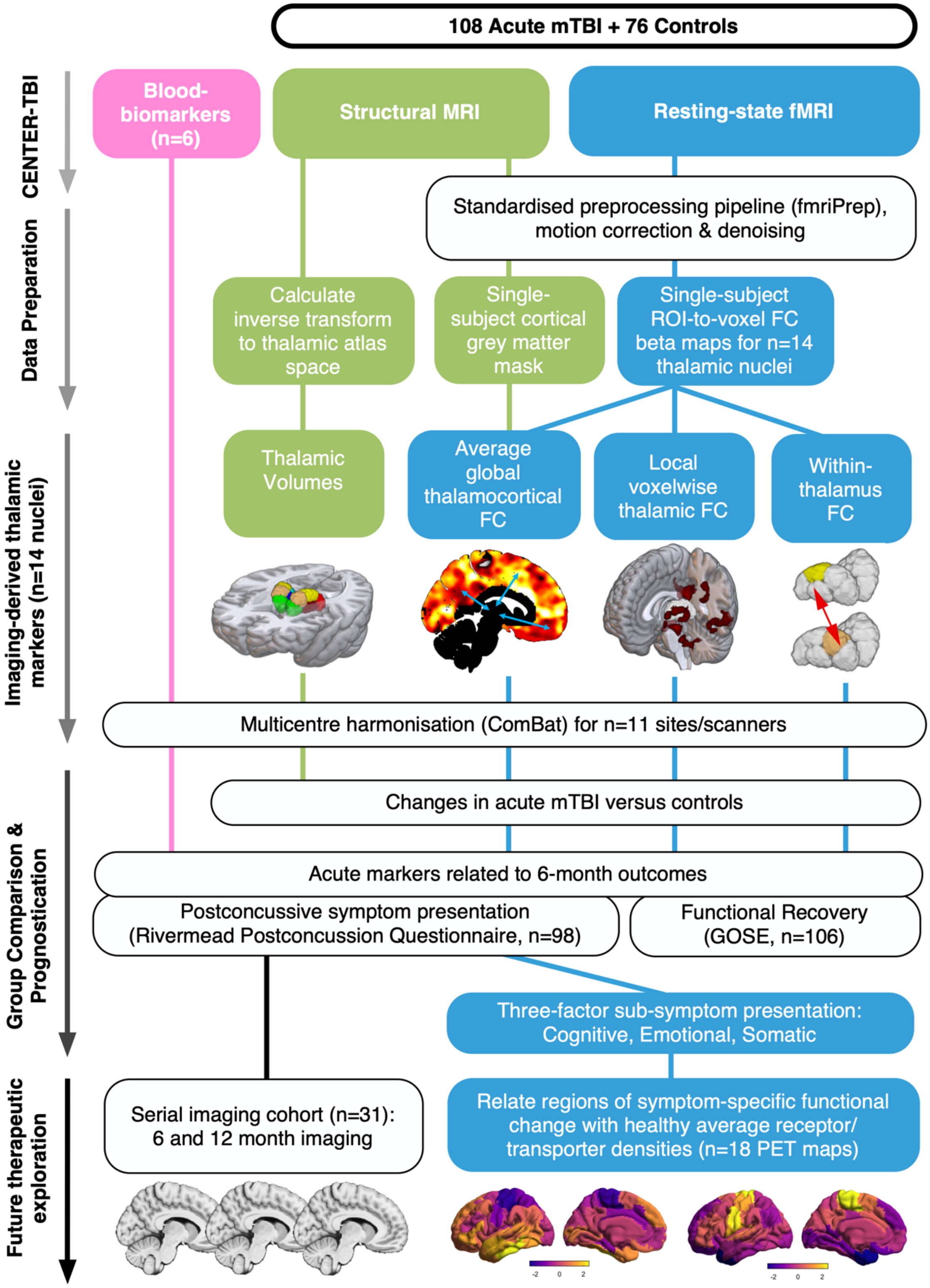

**Table S1. Demographic information for wider cohort of mTBI.** These data are for patients meeting our specified non-imaging inclusion criteria, regardless of whether acute imaging was performed. These are to show the similarity in demographic characteristics between our patient cohort and the wider mTBI population.

| | | **mTBI (n=975)**<br>n (%) |
|---|---|---|
| **Age** | | |
| 18-35 | | 353 (36.2) |
| 36-55 | | 340 (34.9) |
| 55-70 | | 282 (28.9) |
| **Sex** | | |
| Male | | 645 (68.2) |
| Female | | 330 (33.8) |
| **Glasgow Coma Score** | | |
| 15 | | 836 (85.7) |
| 14 | | 124 (12.7) |
| 13 | | 15 (1.5) |
| **Injury Cause** | | |
| Road Traffic Incident | | 369 (39.5) |
| Incidental Fall | | 397 (42.5) |
| Other Non-intentional injury | | 81 (8.7) |
| Violence/Assault | | 85 (9.1) |
| Act of Mass Violence | | 3 (0.3) |
| Unknown | | 44 (4.5) |
| **Strata** | | |
| Emergency Room | | 466 (47.7) |
| Admission | | 509 (52.2) |
| **6 Month GOSE** | | |
| Complete | | 508 (52.1) |
| Incomplete | | 467 (47.9) |
| **6 Month PCS** | **n=572** | |
| PCS+ | | 186 (32.5) |
| PCS- | | 386 (67.5) |

# Materials and Methods

## i) Fmriprep preprocessing boilerplate

The below boilerplate text describing preprocessing pipeline was automatically generated by fMRIPrep with the express intention that users should copy and paste this text into their manuscripts *unchanged*. It is released under the CC0 license. Results included in this manuscript come from preprocessing performed using *fMRIPrep* 1.5.4 *(60)*(RRID:SCR_016216), which is based on *Nipype* 1.3.1 *(61)*(RRID:SCR_002502).

The T1-weighted (T1w) image was corrected for intensity non-uniformity (INU) with N4BiasFieldCorrection *(62)*, distributed with ANTs 2.2.0 *(64)*(RRID:SCR_004757), and used as T1w-reference throughout the workflow. The T1w-reference was then skull-stripped with a *Nipype* implementation of the antsBrainExtraction.sh workflow (from ANTs), using OASIS30ANTs as target template. Brain tissue segmentation of cerebrospinal fluid (CSF), white-matter (WM) and gray-matter (GM) was performed on the brain-extracted T1w using fast (FSL 5.0.9,). Volume-based spatial normalization to one standard space (MNI152NLin2009cAsym) was performed through nonlinear registration with antsRegistration (ANTs 2.2.0), using brain-extracted versions of both T1w reference and the T1w template. The following template was selected for spatial normalization: *ICBM 152 Nonlinear Asymmetrical template version 2009c* [TemplateFlow ID: MNI152NLin2009cAsym].

For each of the 1 BOLD runs found per subject (across all tasks and sessions), the following preprocessing was performed. First, a reference volume and its skull-stripped version were generated using a custom methodology of *fMRIPrep*. Susceptibility distortion correction (SDC) was omitted. The BOLD reference was then co-registered to the T1w reference using flirt (FSL 5.0.9)*(73)* with the boundary-based registration cost-function. Co-registration was configured with nine degrees of freedom to account for distortions remaining in the BOLD reference. Head-motion parameters with respect to the BOLD reference (transformation matrices, and six corresponding rotation and translation parameters) are estimated before any spatiotemporal filtering using mcflirt (FSL 5.0.9)*(73)*. The BOLD time-series (including slice-timing correction when applied) were resampled onto their original, native space by applying the transforms to correct for head-motion. These resampled BOLD time-series will be referred to as *preprocessed BOLD in original space*, or just *preprocessed BOLD*. The BOLD time-series were resampled into standard space, generating a *preprocessed BOLD run in ['MNI152NLin2009cAsym'] space*. First, a reference volume and its skull-stripped version were generated using a custom methodology of *fMRIPrep*. Several confounding time-series were calculated based on the *preprocessed BOLD*: framewise displacement (FD), DVARS and three region-wise global signals. FD and DVARS are calculated for each functional run, both using their implementations in *Nipype* . The three global signals are extracted within the CSF, the WM, and the whole-brain masks. The head-motion estimates calculated in the correction step were also placed within the corresponding confounds file. Frames that exceeded a threshold of 0.5 mm FD or 1.5 standardised DVARS were annotated as motion outliers. All resamplings can be performed with *a single interpolation step* by composing all the pertinent transformations (i.e. head-motion transform matrices, susceptibility distortion correction when available, and co-registrations to anatomical and output spaces).

Many internal operations of *fMRIPrep* use *Nilearn* 0.6.0, mostly within the functional processing workflow. For more details of the pipeline, see the section corresponding to workflows in *fMRIPrep*'s documentation.

### ii) Denoising quality control – subject motion

Higher motion parameters are a common signature of patient populations, and as such should be treated with caution. However, this poses a conflict; we need to avoid spurious motion-induced changes in connectivity estimates whilst still including a representative patient sample which may intrinsically display greater motion. Simply excluding all patients with high motion may be systematically excluding a group of interest and introduce sampling bias.

We therefore compared metrics of number of volumes removed, mean DVARS and mean framewise displacement (FD) between the patient and control cohorts. We additionally correlated these with thalamocortical functional connectivity, to ensure group differences in motion were not attributable to group differences in connectivity.

The mean number of volumes removed in the mTBI cohort (21.60, SD=17.97) was significantly greater that the mean removed volumes in the healthy control cohort (13.79, SD=13.87) ($F_{1,180}$=9.4, $p$=.004). Prior to removal of these volumes and denoising, mean DVARS was higher in the mTBI cohort (27.84, SD=7.69) versus the healthy control cohort (23.82, SD=7.65) ($F_{1,180}$=9.8, $p$=.004), as was mean framewise displacement higher in the mTBI cohort (21.60, SD=17.97) compared to the healthy control cohort (0.19, SD=0.10) ($F_{1,180}$=7.5, $p$=.007). Importantly however, we found no significant correlation between motion and connectivity estimates following denoising in any of the n=16 thalamic seeds, demonstrated in the correlation plot below (p-value as text, r as background colour).

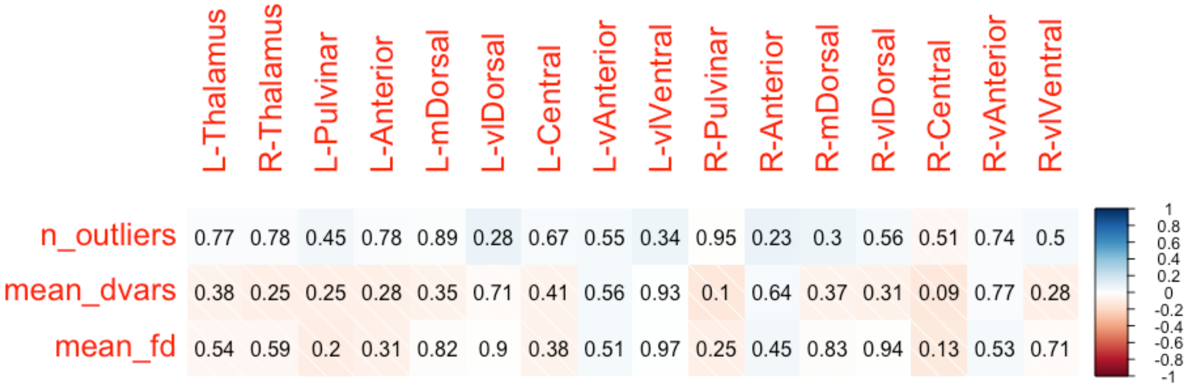

### iii) Harmonization validation

To provide some additional support for the use of Neurocombat harmonization for possible site differences in our multicentre study, we have compared site differences before and after harmonization in two imaging domains; T1w MRI thalamic volume, and rs-fMRI thalamocortical functional connectivity. Replicating methods used in the original NeuroCombat papers, we have used a Kruskal Wallis test on each variable before and after harmonization, to ascertain change in site differences.

Thalamus volume: both before and harmonization, 0/16 nuclei showed significant differences between sites.

Thalamocortical FC: before harmonization, 4/16 (25%) nuclei showed significant differences across the 11 sites. These 4 nuclei were: left pulvinar ($H(10)=38.4$, $p=.002$), left vl-ventral ($H(10)=29.2$, $p=.024$), right pulvinar ($H(10)=40.5$, $p=.002$), and right vl-ventral ($H(10)=29.8$, $p=.024$). None of these 4 nuclei were found to have significant differences between the mTBI and control groups in our manuscript and thus were not explored in further detail. Following harmonization, 0/16 nuclei showed significant site differences, nevertheless demonstrating the success of these methods.

We are therefore confident in the ability of this method to address some of the harmonization issues arising within multicentre acquisition.

**Fig S2. Rivermead postconcussive symptom reporting.** Measured as count per response. Responses to postconcussive items on the RPQ recorded at 6 months post-injury. Score indicates symptom experience compared to pre-injury levels on a five-point scale: 0 = 'not experienced at all', 1 = 'no more of a problem', 2 = 'a mild problem', 3 = 'a moderate problem', 4 = 'a severe problem'.

A. RPQ scores across mTBI cohort (n=98)

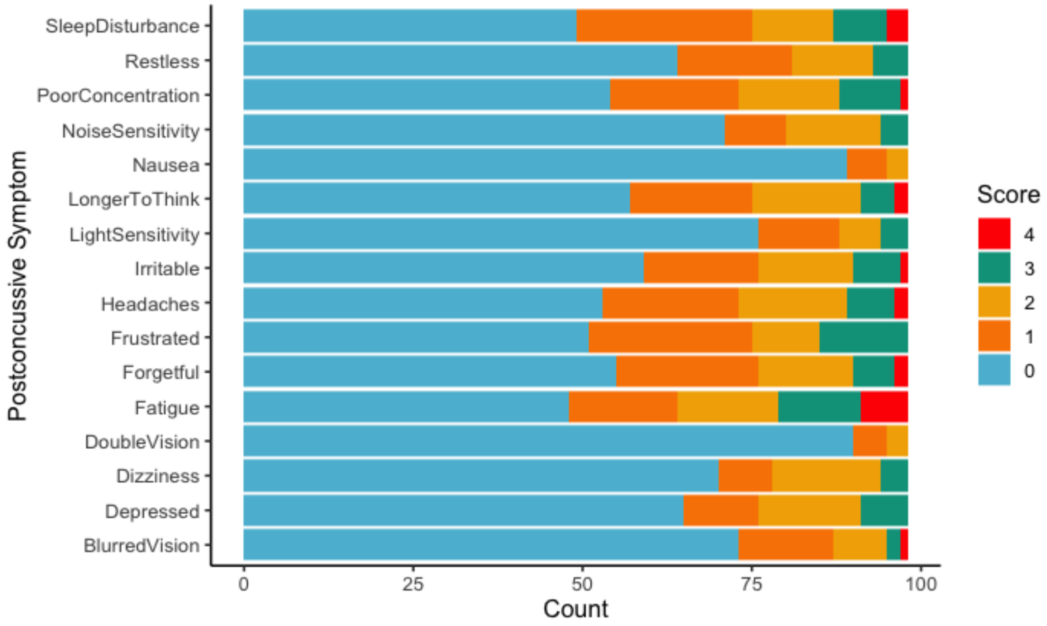


B. RPQ scores across mTBI cohort with three or more symptoms (i.e. PCS+ group; n=31)

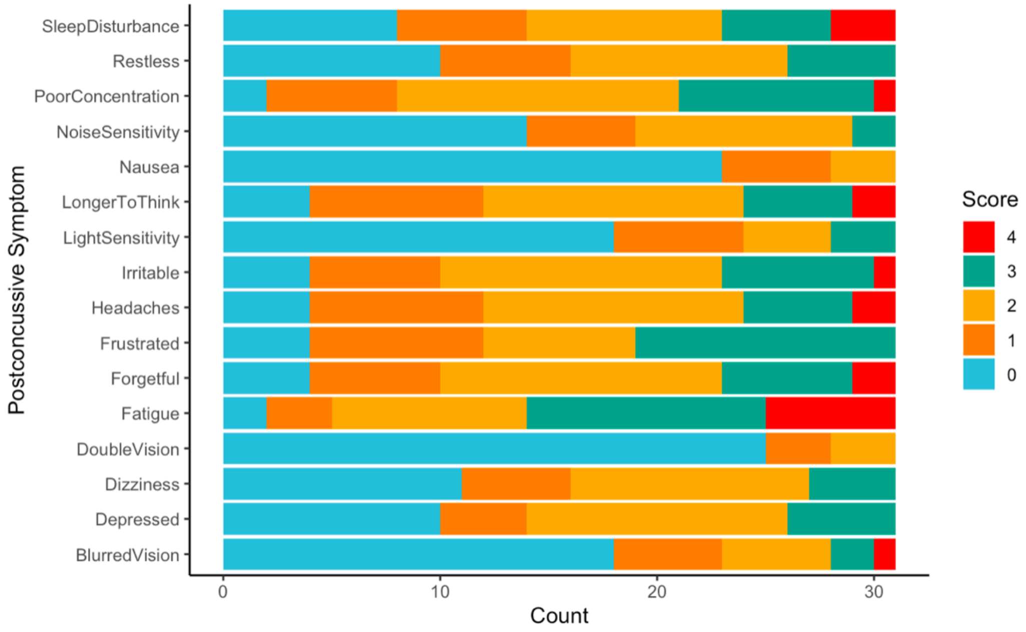

**Fig S3. Change in postconcussive symptoms from baseline to 6-months.** Acute RPQ scores were collected for n=85/98 of the patients with 6-month RPQ scores, at 1.02 ± 1.85 days post-injury. These are compared between the PCS+ and PCS- groups within mTBI.

Total RPQ score at 6 months in this PCS+ group (mean=19.6, SD=9.45) was significantly higher than in the PCS- group (mean=1.89, SD=3.2) using a Mann Whitney U-test ($W = 25$ $p<.001$), as expected. However, total RPQ scores showed no statistical difference between the two groups at baseline (W = 658, p = .19). We present below the change in total RPQ score per patient, color-coded by 6-month PCS group, to show change in postconcussive symptom severity over time.

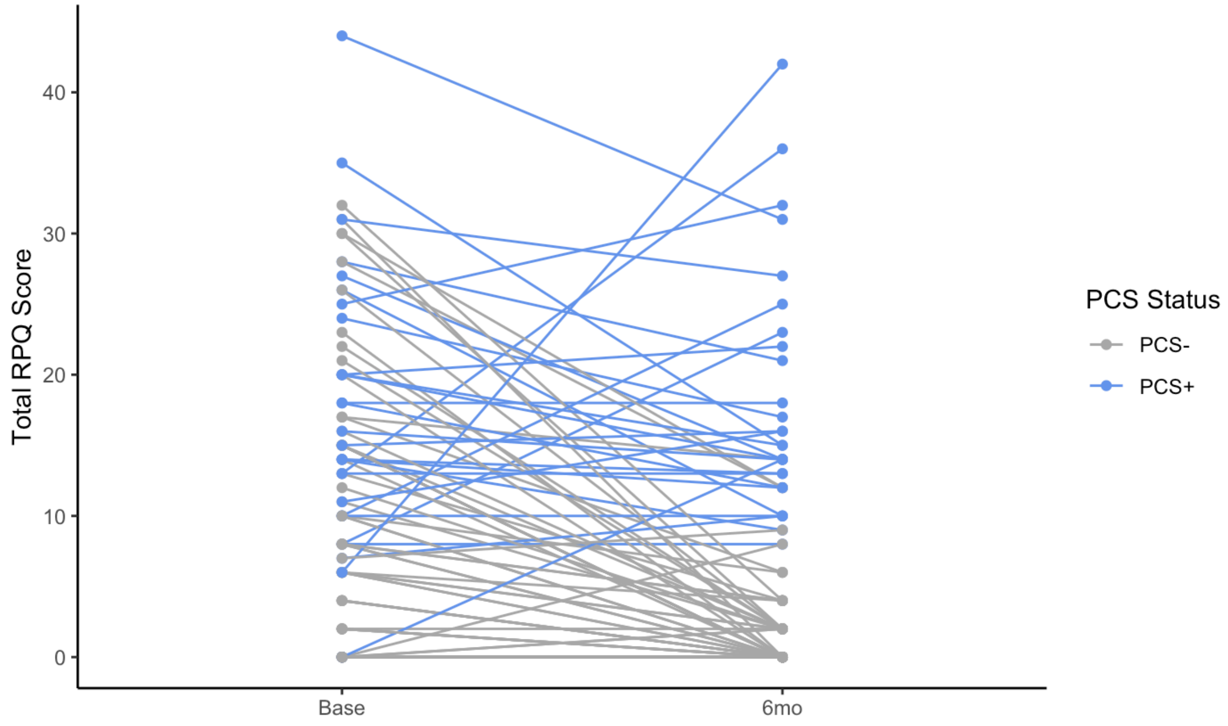

**Table S2. Within-thalamus comparisons of outcome groups**

| Comparison | Test (df) | Average within-thalamus FC | | |
|---|---|---|---|---|
| | | **Left vAnterior** | **Right vAnterior** | **Right vlDorsal** |
| **6-Month GOSE** | | | | |
| GOSE≤7 vs HC | F-test (*1,120*) | *F*=4.4, *p*=.17 | *F*=1.7, *p*=.34 | ***F*=9.0, *p*=.03** |
| GOSE-8 vs HC | F-test (*1,130*) | *F*<0.01, *p*=.99 | *F*<0.01, *p*=.99 | *F*=0.9, *p*=.53 |
| GOSE≤7 vs GOSE-8 | F-test (*1,100*) | *F*=1.9, *p*=.34 | *F*=0.3, *p*=.73 | *F*=2.0, *p*=.34 |
| **6-Month PCS** | | | | |
| PCS+ vs HC | F-test (*1,103*) | *F*=2.8, *p*=.43 | *F*=1.8, *p*=.46 | *F*=6.7, *p*=.10 |
| PCS- vs HC | F-test (*1,139*) | *F*=0.1, *p*=.84 | *F*<0.01, *p*=.99 | *F*=1.6, *p*=.46 |
| PCS+ vs PCS- | F-test (*1,92*) | *F*=0.1, *p*=.84 | *F*=0.2, *p*=.84 | *F*=1.3, *p*=.46 |

HC= Healthy Controls. All p-values shown are FDR-corrected. Bold indicates statistical significance at p<0.05, whereby GOSE≤7 group had higher FC than HC.

**Fig S4. Symptom-specific outcome groups' thalamocortical connectivity**

In symptom-specific outcome groups, patients were split into groups with or without cognitive (Cog+ n=38; Cog- n=60), emotional (Emo+ n=38; Emo- n=60), or somatic symptoms (Som+ n=23; Som- n=75). Although the cognitive and emotional groups have identical group numbers, they do not comprise identical patients. There is, nevertheless, a high overlap of these groups which have a significant association ($X^2$1=42, p<.001).

To answer whether specific subgroups might be driving group effects of higher thalamocortical connectivity, we have plotted here the 4 possible groups; presenting no chronic symptoms (n=52), only cognitive (n=8), only emotional (n=8), and both cognitive and emotional symptoms (n=30).

Importantly, a between-subjects ANOVA between the three possible subject groups with symptoms shows no statistical differences in the L-vAnterior ($F_{1,2}$=0.55, $p$=.68), R-vlDorsal ($F_{1,2}$=0.38, $p$=.68), or R-vAnterior ($F_{1,2}$=0.56, $p$=.68). These tests additionally included covariates of sex, age, time since injury, and initial GCS, and are corrected for multiple comparisons. Thus, presenting both cognitive and emotional symptoms concurrently is not significantly different to either individually in this small cohort.

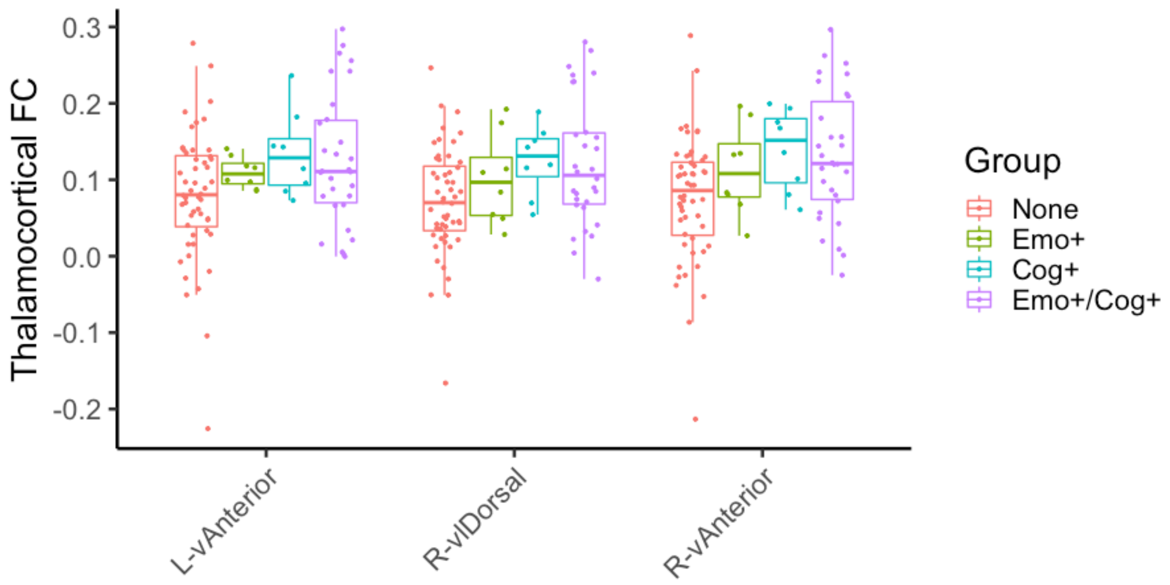

**Fig S5. Network involvement of voxelwise results.** Wedge plots of spatial overlap between voxels surviving cluster correction in thalamic voxelwise connectivity tests between outcome groups and canonical intrinsic connectivity networks (ICN). These networks are defined by ICN_atlas toolbox as an extension to SPM using the ICN-BM atlas *(32)*. Colour bars indicate functional relevance. These are not statistically tested, merely a visual aid for relative functional involvement of SPM-t maps.

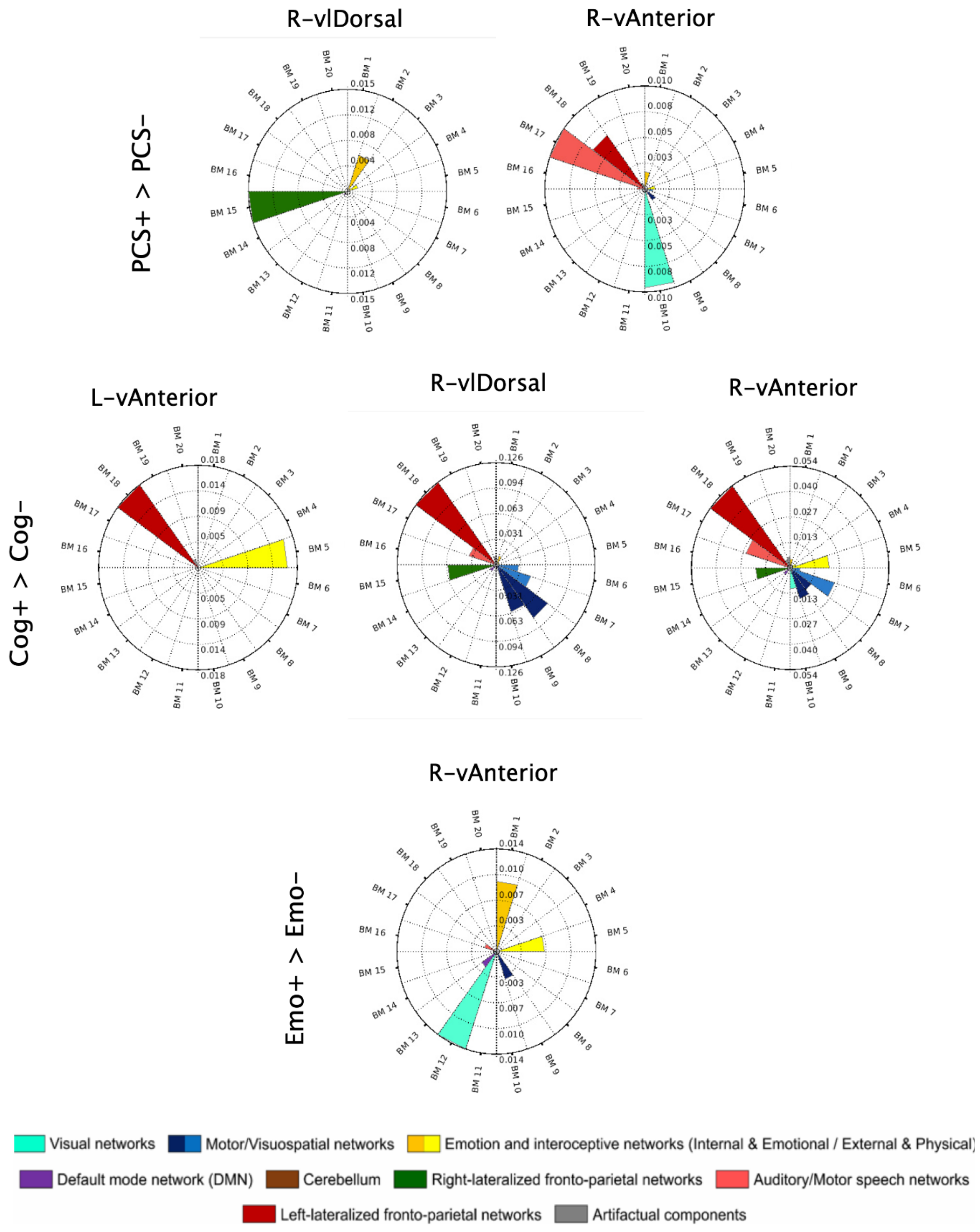

**Table S3. Additional correlations to PET maps.** All correlations between investigated seed-to-voxel t-maps and z-scored PET maps. Values shown are from the Schaefer 200 Parcellation after FDR correction, with significant associations in bold. Cells with no data indicate this correlation was not calculated as significance was not found at the mTBI-HC level. Asterisk indicates this correlation was significant in the Glasser360 parcellation (p=0.007).

| PET map | mTBI > HC | | | Cog+ > Cog- | | | Emo+ > Emo- |
|---|---|---|---|---|---|---|---|
| | L-vAnterior | R-vlDorsal | R-vAnterior | L-vAnterior | R-vlDorsal | R-vAnterior | R-vAnterior |
| 5HT-1A | r=-0.1, p=0.41 | r=0.18, p=0.13 | r=-0.28, p=0.24 | - | - | - | - |
| 5HT-1B | r=-0.09, p=0.4 | r=0.17, p=0.14 | r=0, p=0.57 | - | - | - | - |
| 5HT-2A | **r=-0.38, p=0.01** | r=-0.19, p=0.13 | **r=-0.5, p=0.006** | r=-0.05, p=0.40 | - | r=-0.16, p=0.31 | **r=-0.14, p=0.06*** |
| 5HT-4 | r=-0.31, p=0.11 | r=-0.11, p=0.34 | **r=-0.47, p=0.02** | - | - | r=-0.15, p=0.35 | r=-0.12, p=0.07 |
| 5HT-6 | r=0.01, p=0.49 | **r=0.28, p=0.003** | r=-0.11, p=0.43 | - | r=0.19, p=0.32 | - | - |
| 5HTT | r=0.05, p=0.45 | r=0.12, p=0.25 | r=-0.23, p=0.35 | - | - | - | - |
| $\alpha 4\beta 2$ | r=-0.16, p=0.34 | r=0.19, p=0.12 | r=-0.02, p=0.51 | - | - | - | - |
| CB1 | r=-0.31, p=0.12 | r=0, p=0.56 | r=-0.36, p=0.13 | - | - | - | - |
| D1 | r=0.12, p=0.39 | **r=0.31, p<0.001** | r=-0.03, p=0.49 | - | r=-0.10, p=0.46 | - | - |
| D2 | r=-0.13, p=0.39 | r=0.17, p=0.18 | r=-0.31, p=0.19 | - | - | - | - |
| DAT | r=0.18, p=0.32 | **r=0.4, p<0.001** | r=-0.01, p=0.51 | - | r=0.01, p=0.47 | - | - |
| GABA-A | r=-0.01, p=0.54 | r=0.02, p=0.45 | r=-0.08, p=0.43 | - | - | - | - |
| H3 | r=-0.14, p=0.37 | r=0.17, p=0.14 | r=-0.12, p=0.4 | - | - | - | - |
| M1 | r=-0.07, p=0.4 | r=0.07, p=0.38 | r=-0.11, p=0.34 | - | - | - | - |
| mGluR5 | r=-0.07, p=0.43 | **r=0.30, p=0.01** | r=-0.16, p=0.35 | - | r=0.12, p=0.40 | - | - |
| MU | r=-0.33, p=0.12 | r=0.09, p=0.37 | r=-0.32, p=0.19 | - | - | - | - |
| NAT | **r=0.39, p=0.006** | **r=0.49, p<0.001** | **r=0.41, p=0.02** | **r=0.31, p=0.003** | **r=0.49, p<0.001** | **r=0.43, p=0.006** | **r=0.24, p=0.006** |
| VAChT | r=0.23, p=0.18 | **r=0.47, p<0.001** | r=0.09, p=0.41 | - | **r=0.40, p<0.001** | - | - |

**Table S4. Longitudinal thalamic volume comparisons.** FDR-corrected results from groups comparisons of thalamic nuclei volume, corrected for sex and age. HC = healthy controls. First results column details comparisons between controls and acute volumes of the longitudinal cohort (n=31). Second column details within-subjects ANOVA of volume change over time (acute, 6mo, 12mo) in this longitudinal cohort.

| **Thalamic ROI** | **mTBI vs HC**<br>**F-test (*1,104*)** | **mTBI over time**<br>**F-test (2,*48*)** |
|---|---|---|
| Left Thalamus | *F*=1.3, *p*=.68 | *F*=2.7, *p*=.52 |
| Right Thalamus | *F*=1.5, *p*=.68 | *F*=1.9, *p*=.52 |
| **Left-hemisphere nuclei** | | |
| Pulvinar | *F*=0.04, *p*=.87 | *F*=1.9, *p*=.52 |
| Anterior | *F*=2.2, *p*=.68 | *F*=0.2, *p*=.86 |
| mDorsal | *F*=0.5, *p*=.73 | *F*=1.6, *p*=.54 |
| vlDorsal | *F*=0.7, *p*=.71 | *F*=0.3, *p*=.86 |
| Central | *F*=0.6, *p*=.71 | *F*=0.3, *p*=.86 |
| vAnterior | *F*=5.9, *p*=.26 | *F*=0.2, *p*=.86 |
| vlVentral | *F*=0.1, *p*=.87 | *F*=1.1, *p*=.67 |
| **Right-hemisphere nuclei** | | |
| Pulvinar | *F*=0.03, *p*=.87 | *F*=0.2, *p*=.86 |
| Anterior | *F*=0.9, *p*=.71 | *F*=0.5, *p*=.86 |
| mDorsal | *F*=0.7, *p*=.71 | *F*=2.0, *p*=.52 |
| vlDorsal | *F*=1.7, *p*=.68 | *F*=1.5, *p*=.54 |
| Central | *F*=0.03, *p*=.87 | *F*=2.6, *p*=.52 |
| vAnterior | *F*=3.7, *p*=.46 | *F*=0.06, *p*=.94 |
| vlVentral | *F*=0.09, *p*=.87 | *F*=0.4, *p*=.86 |

**Fig S6. Longitudinal change of hyperconnected clusters.** All data show distribution of mean beta value within hyperconnected clusters defined in the mTBI-control comparison (main text figure 2). Top: Mixed ANOVA between acute and 12mo timepoints between groups, where p-values given are interaction effects between timepoint (acute or 12mo) and group (PCS+ or PCS-). Shaded regions give the IQR of controls for each nucleus, with solid line indicating the controls' mean. Bottom: Post-hoc results within-subjects finding significant decreases in FC only in those with PCS. Lines join individual subjects' data.

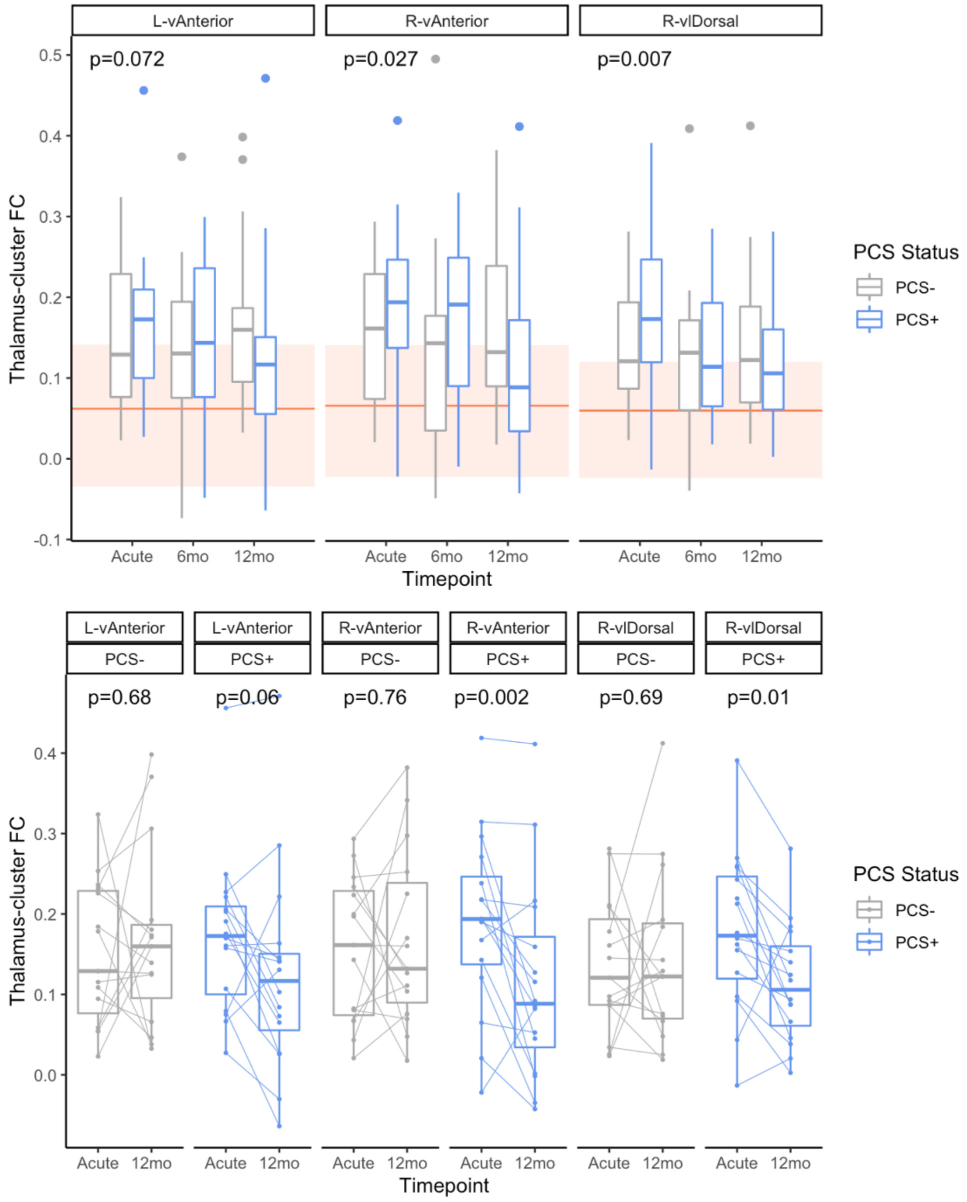

**Figure S7. Thalamic nuclei masks.** Colour shows bilateral thalamic 'group' whereby labelling indicates a known anatomical nucleus or nuclei group, with the exception of the 'Central' group which represents the central lateral, lateral posterior, and some anterior medial pulvinar. Thalamic groups were analysed in each hemisphere individually, to form 7 subdivisions per hemisphere. Abbreviations: vAnterior (ventral anterior group), vlDorsal (ventral lateral posterior, dorsal division group), mDorsal (medial dorsal group), vlVentral (ventral-lateral ventral group). Derived from Najdenovska and colleagues.

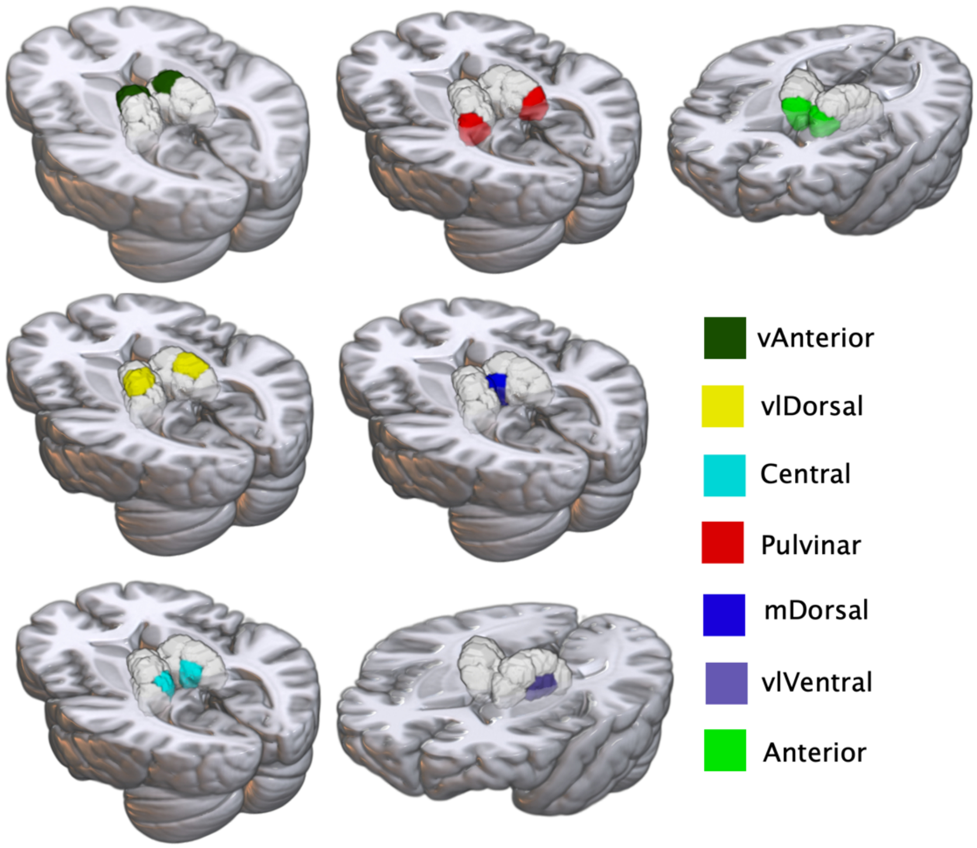

Supplement: awad056_Supplementary_Data [file awad056_supplementary_data.zip › brain-2022-01916-File010.pdf]
