# Supplementary material for: Acute thalamic connectivity precedes chronic post-concussive symptoms in mild traumatic brain injury
Source: Brain. 2023 Feb 22;146(8):3484–99. doi: 10.1093/brain/awad056 (PMC10393415; doi:10.1093/brain/awad056)
Supplement: awad056_Supplementary_Data [file awad056_supplementary_data.zip › brain-2022-01916-File011.pdf]

Additional groups and authors

CENTER-TBI MRI Substudy Participants and Investigators: Krisztina Amrein, MD, János Szentágothai Research Centre, University of Pécs, Pécs, Hungary; Nada Andelic, MD, PhD, Division of Surgery and Clinical Neuroscience, Department of Physical Medicine and Rehabilitation, Oslo University Hospital and University of Oslo, Oslo, Norway; Lasse Andreassen, MD, Department of Neurosurgery, University Hospital Northern Norway, Tromso, Norway; Audny Anke, MD, Department of Physical Medicine and Rehabilitation, University Hospital Northern Norway, Tromso, Norway; Philippe Azouvi, MD, PhD, Raymond Poincare Hospital, Assistance Publique–Hopitaux de Paris (AP-HP), Paris, France; Bo-Michael Bellander, MD, PhD, Department of Neurosurgery and Anesthesia and Intensive Care Medicine, Karolinska University Hospital, Stockholm, Sweden; Habib Benali, MD, Anesthesie-Réanimation, AP-HP, Paris, France; Andras Buki, DSc, Department of Neurosurgery, Medical School, and Neurotrauma Research Group, János Szentágothai Research Centre, University of Pécs, Pécs, Hungary; Alessio Caccioppola, MD, Neuro Intensive Care Unit (ICU), Fondazione Istituto di Ricovero e Cura a Carattere Scientifico (IRCCS) Cà Granda Ospedale Maggiore Policlinico, Milan, Italy; Emiliana Calappi, MD, Neuro ICU, Fondazione IRCCS Cà Granda Ospedale Maggiore Policlinico, Milan, Italy; Marco Carbonara, MD, Neuro ICU, Fondazione IRCCS Cà Granda Ospedale Maggiore Policlinico, Milan, Italy; Giuseppe Citerio, MD, PhD, School of Medicine and Surgery, Università Milano Bicocca, Milan, Italy; and NeuroIntensive Care, Azienda Socio Sanitaria Territoriale di Monza, Monza, Italy; Hans Clusmann, MD, Department of Neurosurgery, Medical Faculty Rheinisch-Westfälische Technische Hochschule Aachen University, Aachen, Germany; Mark Coburn, MD, Department of Anaesthesiology, University Hospital of Aachen, Aachen, Germany, and Department of Anesthesiology and Intensive Care Medicine, University Hospital Bonn, Bonn, Germany; Jonathan Coles, MD, PhD, Department of Anesthesia and Neurointensive Care, Cambridge University Hospital NHS Foundation Trust, Cambridge, United Kingdom; Marta Correia, PhD, Radiology/MRI department, MRC (Medical Research Council) Cognition and Brain Sciences Unit, Cambridge, United Kingdom; Endre Czeiter, PhD, Department of Neurosurgery, Medical School, and Neurotrauma Research Group, János Szentágothai Research Centre, University of Pécs, Pécs, Hungary; Véronique De Keyser, MSc, Department of Neurosurgery, Antwerp University Hospital and University of Antwerp, Edegem, Belgium; Vincent Degos, MD, Anesthesie-Réanimation, AP-HP, Paris, France; Bart Depreitere, MD, PhD, Department of Neurosurgery, University Hospitals Leuven,

Leuven, Belgium; Live Eikenes, PhD, Department of Circulation and Medical Imaging, Norwegian University of Science and Technology (NTNU), Trondheim, Norway; Erzsébet Ezer, MD, Department of Anaesthesiology and Intensive Therapy, University of Pécs, Pécs, Hungary; Kelly Foks, MD, PhD, Department of Neurology, Erasmus MC, Rotterdam, the Netherlands; Shirin Frisvold, Department of Anesthesiology and Intensive Care, University Hospital Northern Norway, Tromso, Norway; Damien Galanaud, MD, Anesthesie-Réanimation, AP-HP, Paris, France; Alexandre Ghuysen, MD, Emergency Department, Centre Hospitalier Universitaire, Liège, Belgium; Ben Glocker, PhD, Department of Computing, Imperial College London, London, United Kingdom; Asta Haberg, PhD, Department of Neuromedicine and Movement Science, NTNU, and Department of Physical Medicine and Rehabilitation, St Olavs Hospital, Trondheim University Hospital, Trondheim, Norway; Iain Haitsma, MD, Department of Neurosurgery, Erasmus MC, Rotterdam, the Netherlands; Eirik Helseth, Department of Neurosurgery, Oslo University Hospital, Oslo, Norway; Peter J. Hutchinson, MD, PhD, Division of Neurosurgery, Department of Clinical Neurosciences, Addenbrooke's Hospital and University of Cambridge, Cambridge, United Kingdom; Evgenios Kornaropoulos, PhD, Division of Anaesthesia, University of Cambridge, Addenbrooke's Hospital, Cambridge, United Kingdom; Noémi Kovács, PhD, Hungarian Brain Research Program, University of Pécs, Pécs, Hungary; Ana Kowark, MD, Department of Anaesthesiology, University Hospital of Aachen, Aachen, Germany; Steven Laureys, MD, PhD, Cyclotron Research Center, University of Liège, Liège, Belgium; Didier Ledoux, MD, PhD, Cyclotron Research Center, University of Liège, Liège, Belgium; Hester Lingsma, PhD, Department of Public Health, Erasmus Medical Center–University Medical Center, Rotterdam, the Netherlands; Andrew I. R. Maas, MD, PhD, Department of Neurosurgery, Antwerp University Hospital and University of Antwerp, Edegem, Belgium; Geoffrey Manley, MD, PhD, Department of Neurological Surgery, University of California, San Francisco; David K. Menon, MD, PhD, Division of Anaesthesia, University of Cambridge, Addenbrooke's Hospital, Cambridge, United Kingdom; Tomas Menovsky, MD, PhD, Department of Neurosurgery, Antwerp University Hospital and University of Antwerp, Edegem, Belgium; Benoit Misset, MD, Cyclotron Research Center, University of Liège, Liège, Belgium; Visakh Muraleedharan, MSc, Karolinska Institutet, International Neuroinformatics Coordinating Facility, Stockholm, Sweden; Ingeborg Nakken, MSc, Department of Radiology and Nuclear Medicine, St Olavs Hospital, Trondheim University Hospital, Trondheim, Norway; Virginia Newcombe, MD, PhD, Division of Anaesthesia, University of Cambridge, Addenbrooke's Hospital, Cambridge, United Kingdom; Wibeke Nordhøy, PhD, Department of Diagnostic

Physics, Clinic of Radiology and Nuclear Medicine, Oslo University Hospital, Oslo, Norway; József Nyirádi, PhD, János Szentágothai Research Centre, University of Pécs, Pécs, Hungary; Fabrizio Ortolano, MD, Neuro ICU, Fondazione IRCCS Cà Granda Ospedale Maggiore Policlinico, Milan, Italy; Paul M. Parizel, MD, PhD, David Hartley Chair of Radiology, Royal Perth Hospital and University of Western Australia, Perth, Australia; Vincent Perlbarg, PhD, Anesthesie-Réanimation, AP-HP, Paris, France; Paolo Persona, MD, Department of Anesthesia and Intensive Care, Azienda Ospedaliera Università di Padova, Padova, Italy; Wilco Peul, MD, PhD, Department of Neurosurgery, Leiden University Medical Center, Leiden, and Department of Neurosurgery, Medical Center Haaglanden, The Hague, the Netherlands; Jussi P. Posti, MD, PhD, Division of Clinical Neurosciences, Department of Neurosurgery and Turku Brain Injury Centre, Turku University Hospital and University of Turku, Turku, Finland; Louis Puybasset, MD, PhD, Department of Anesthesiology and Critical Care, Pitié -Salpêtrière Teaching Hospital, AP-HP, and University Pierre et Marie Curie, Paris, France; Sophie Richter, MD, Division of Anaesthesia, University of Cambridge, Addenbrooke's Hospital, Cambridge, United Kingdom; Cecilie Roe, MD, Department of Physical Medicine and Rehabilitation, Oslo University Hospital/University of Oslo, Oslo, Norway; Olav Roise, MD, Division of Orthopedics, Oslo University Hospital, and Institute of Clinical Medicine, Faculty of Medicine, University of Oslo, Oslo, Norway; Rolf Rossaint, MD, Department of Anaesthesiology, University Hospital of Aachen, Aachen, Germany; Sandra Rossi, MD, Department of Anesthesia and Intensive Care, Azienda Ospedaliera Università di Padova, Padova, Italy; Daniel Rueckert, PhD, Department of Computing, Imperial College London, London, United Kingdom; Toril Skandsen, MD, PhD, Department of Neuromedicine and Movement Science, NTNU, and Department of Physical Medicine and Rehabilitation, St Olavs Hospital, Trondheim University Hospital, Trondheim, Norway; Abayomi Sorinola, MD, Department of Neurosurgery, University of Pécs, Pécs, Hungary; Emmanuel Stamatakis, PhD, Division of Anaesthesia, University of Cambridge, Addenbrooke's Hospital, Cambridge, United Kingdom; Ewout W. Steyerberg, PhD, Department of Public Health, Erasmus Medical Center–University Medical Center, Rotterdam, and Department of Biomedical Data Sciences, Leiden University Medical Center, Leiden, the Netherlands; Nino Stocchetti, MD, Department of Pathophysiology and Transplantation, Milan University, and Neuroscience ICU, Fondazione IRCCS Cà Granda Ospedale Maggiore Policlinico, Milano, Italy; Riikka Takala, MD, PhD, Perioperative Services, Intensive Care Medicine and Pain Management, Turku University Hospital and University of Turku, Turku, Finland; Viktória Tamás, MD, Department of Neurosurgery, University of Pécs, Pécs, Hungary; Olli Tenovuo, MD, PhD, Department of

Clinical Neurosciences and Turku Brain Injury Centre, Turku University Hospital and University of Turku, Turku, Finland; Zoltán Vámos, MD, Department of Anaesthesiology and Intensive Therapy, University of Pécs, Pécs, Hungary; Gregory Van der Steen, MSc, Department of Neurosurgery, Antwerp University Hospital and University of Antwerp, Edegem, Belgium; Wim Van Hecke, PhD, icometrix, Leuven, Belgium; Thijs Vande Vyvere, PhD, icometrix, Leuven, Belgium; Jan Verheyden, MSc, icometrix, Leuven, Belgium; Anne Vik, MD, PhD, Department of Neuromedicine and Movement Science, NTNU, and Department of Neurosurgery, St Olavs Hospital, Trondheim University Hospital, Trondheim, Norway; Victor Volovici, MD, PhD, Department of Neurosurgery, Erasmus MC, Rotterdam, the Netherlands; Lars T. Westlye, PhD, Norwegian Centre for Mental Disorders Research, Division of Mental Health and Addiction, Oslo University Hospital and Institute of Clinical Medicine, University of Oslo, and Department of Psychology, University of Oslo, Oslo, Norway; Guy Williams, PhD, Division of Anaesthesia, University of Cambridge, Addenbrooke's Hospital, Cambridge, United Kingdom; Stefan Winzeck, MSc, Division of Anaesthesia, University of Cambridge, Addenbrooke's Hospital, Cambridge, United Kingdom; Peter Ylén, PhD, VTT Technical Research Centre, Tampere, Finland; and Tommaso Zoerle, MD, Neuro ICU, Fondazione IRCCS Cà Granda Ospedale Maggiore Policlinico, Milan, Italy.
